# Compost application boosts soil restoration in highly disturbed hillslope vineyard

**DOI:** 10.3389/fpls.2023.1289288

**Published:** 2023-11-23

**Authors:** Marco Lucchetta, Alessandro Romano, Monica Yorlady Alzate Zuluaga, Flavio Fornasier, Sonia Monterisi, Youry Pii, Patrick Marcuzzo, Lorenzo Lovat, Federica Gaiotti

**Affiliations:** ^1^ Council for Agricultural Research and Economics-Research Centre for Viticulture and Enology (CREA-VE), Conegliano, TV, Italy; ^2^ Faculty of Agricultural, Environmental and Food Sciences, Free University of Bozen/Bolzano, Bolzano, Italy; ^3^ Council for Agricultural Research and Economics-Research Centre for Viticulture and Enology (CREA-VE), Gorizia, Italy

**Keywords:** compost, enzyme activity, grapevine, metagenomics, soil microbiome, vineyards

## Abstract

A field trial was carried out to investigate the effects of compost application on a young Cabernet sauvignon vineyard located in a hilly area in the North-East of Italy and subjected to land terracing before plantation. The use of a compost based on manure, pruning residues and pomace at a rate of 65 t ha^-1^ was compared to the mineral fertilization regime recommended for the vineyards in the area (NPK: 80, 50, 200 kg ha^-1^). A multi-factorial approach that considered soil chemical properties, microbial community structure and function, vine nutritional and vegetative indexes, yield and quality parameters was applied in the attempt of depict interrelated effects of compost on all these factors. Results of this study show that the application of compost for three consequent years greatly increased soil organic matter content and improved the mineral nutrient availability in the soil. Soil biological fertility showed a slow but significant response to compost addition as from the second year of treatment microbial growth and enzyme activity were increased compared to those of the inorganic fertilization, with special regard to enzymes involved in P cycle. A shift in the soil microbial community structure was also observed in compost-treated soil, with higher presence of copiotrophic bacteria, indicators of soil quality, and phosphorus solubilizing bacteria. A decrease of pathogenic fungal strains was also observed. Organic fertilization increased plant nutrient uptake and vegetative growth compared to those observed in chemically fertilized vines. A trend toward increased yield and improvements for some grape quality parameters such as acidity and pH were observed in the first year of production. These results provide evidence that compost can boost soil fertility restoration in vineyard disturbed by land terracing, allowing for agronomic performances comparable or even improved than those of chemically fertilized vines.

## Introduction

Plant nutrition management is crucial for plant growth and development and to sustain quality and production in horticultural crops (Nicholas, 2004). Since the mid-1900s, the use of synthetic fertilizers has increased by about 800% (Mbow et al., 2019), enabling an astounding growth in the yield of numerous crops, including grapevine (Arneth and Denton, 2023). Despite providing enormous benefits for the agricultural industry, the intensive use of chemical solutions for crop nutrition has led to serious downside effects for the environment, impacting air, water, and soil quality. Among them, the use of synthetic nitrogen fertilizers has resulted in a steady increase of N_2_O emissions into the atmosphere over the past decades (Thompson et al., 2019). Fertilizers, when overused, have also an impact on groundwater and inland water bodies through their leaching or running-off (Khan et al., 2017). Fertilization management also plays a major role on soil fertility and productivity. Continuous and excessive fertilizer application can drastically impact soil systems, accelerating acidification and inducing alteration of the main nutrient cycles (Birkhofer et al., 2008). Changes in soil properties may in turn impact the microbial structure of the soil. Studies comparing the effect of environmental and agronomic factors on the soil microbial community composition have shown that the use of chemical NPK fertilizers may represent a disturbance for the soil microorganisms, particularly when the fertilization is continued over several years (Allison and Martiny, 2008). Meanwhile, soil acidification under elevated N inputs could cause soil microbial diversity loss, especially for bacteria, as they display narrower pH ranges for optimal growth compared to fungi (Zhalina et al., 2015; Song et al., 2023).

Soil fertility preservation takes particular priority in hilly areas, as hillside soil are more prone to risk of nutrient loss, alteration of soil profile and structure, and reduction of organic matter due to either erosion or run-off processes (Tarolli and Straffelini, 2020). Grapevine cultivation in sloping soils is very widespread all over the world. In Italy hillside vineyards make up 57% of the total national vineyard surface (Istituto Nazionale di Statistica (ISTAT), 2010), and form part of the most famous wine areas in this Country (Matchplat, 2021). Grape-growing in hilly areas often require substantial intervention on soils, such as incorrect land levelling and terracing, which further contribute to increase the risk of soil degradation and fertility loss (Tarolli et al., 2014). In these contexts, the adoption of fertilization strategies able to maintain crop production while limiting soil degradation is of upmost importance.

The application of compost or other organic input materials is regarded as one of the most potentially viable options for sustainable fertilization management, in compliance with the European Green Deal 2030 guidelines, which among their goals aim to reduce the use of chemical fertilizers by at least 20% by 2030 (Commission, 2020). Plenty of literature agreed to define compost as an excellent soil improver, capable to enhance soil physical, chemical and biological fertility (Bedada et al., 2014; Illera-Vives et al., 2015; Abujabhah et al., 2016; Luna et al., 2016; Baldi et al., 2018). Beside the ameliorating effects on soil quality, several field trials performed on annual and perennial crops showed that compost amendments could also guarantee yield and quality comparable or even improved compared to those achieved with the use of chemical fertilizers (Bedada et al., 2014; Illera-Vives et al., 2015; Baldi et al., 2018).

In vineyards, the use of compost is a widely adopted method for vine nutrition management. In recent years its application has been further boosted by the rising interest of consumers for environmentally-friendly wines, and by the concurrent increase in vineyards converted to organic production (International Organisation of Vine and Wine (OIV), 2019; Masotti et al., 2022).

The effectiveness of compost as a nutrient supplier in vineyards has been well documented for different soils, climate conditions and for several source materials (Nendel and Reuter, 2007; Bustamante et al., 2011; Garzón et al., 2011). Increases in soil organic matter content (SOM) from compost addition account for most of the benefits on soil biological, chemical, and physical properties extensively reported in these and several other studies (Aggelides and Londra, 2000; Abujabhah et al., 2016; Aranyos et al., 2016; Liu et al., 2020). As a source of nutrients for microorganisms, the application of compost typically increases the soil microbial biomass and improves its activity with beneficial effects to the nutrient cycling (Bustamante et al., 2011; Rubio et al., 2013; Lazcano et al., 2020). In light of this, SOM, microbial biomass and enzyme activity of microorganisms are parameters commonly used to assess the effect of compost or other soil management practices on soil fertility.

Compost application has been shown to also affect the composition of the soil microbial community (Allison and Martiny, 2008; Mackie et al., 2015). In a field experiment exploring the effects of biochar and compost in a vineyard in Switzerland, Mackie et al. (2015) found that besides promoting nutrients availability, compost also affected the structure of soil microbial communities, increasing the biodiversity. Results showed that abundance of specific phylum, such as Firmicutes, increased after the application of compost. (Calleja-Cervantes et al., 2015) studied the effect of application of composts from different organic wastes in a vineyard in La Rioja (Spain). They observed changes in the bacterial community for some compost treatments, with increased presence of *Flavobacterium*, *Pedobacter* and *Adhaeribacter* (phylum Bacteroidetes), which typically contribute to improve soil quality. Overall, results from these studies agree pointing out that compost amendments promote large and diverse microbial community which can be a hallmark of healthy soils.

Previous literature reported contrasting results on the impact of compost application on vine growth and yield, as well as on grape quality (total soluble solids, titratable acidity, pH),. (Morlat, 2008; Mugnai et al., 2012) found no significant or small effects on yield and quality after compost amendment to vineyards. Rubio et al. (2013), Gaiotti et al. (2017), Ramos, (2017), Marín-Martínez et al. (2021) reported increased yields and/or pruning weights, as a result of improved soil water and N availability promoted by the compost.

To date, research on the use of organic amendments in vineyards has focused mainly on changes induced on soil chemical and physical characteristics and on the effects on grape yield and quality. Despite the growing interest in the role of the soil microbiome for grapevine nutrition and wine quality, few studies have explored soil biota as part of the complex plant-soil system in vineyards amended with compost. Most of the previous literature documented effects on the microbial biomass, composition and/or enzyme activity. However, interrelated dynamics between soil physical and chemical properties, fungal and bacterial community structure and activity are still poorly explored. Furthermore, relationships between soil and microbial characteristics, plant nutritional status and agronomic performances needs to be simultaneously addressed to better understand the mechanisms underlying belowground ecosystem processes in amended vineyards.

In this study a field trial was established to investigate the effects of application of compost produced from manure, pruning residues and pomace on a Cabernet sauvignon vineyard located in a hilly area in the North-East of Italy and subjected to land terracing before plantation. We addressed the specific goals of: i) investigating the amendment effects on the physico-chemical properties of the soil; ii) explore how the compost addition could shift soil bacterial and fungal structure and enzyme activity; 3) link soil and biota analysis to grapevine nutritional status and agronomic responses. By means of a multi-factorial approach integrating soil-microbiome-plant analysis, this study aims to provide further understanding on the use of compost in vineyard ecosystems.

## Materials and methods

### Study site and treatments

The experiment was carried out during three subsequent growing seasons (2019-2020-2021) in a newly planted Cabernet Sauvignon vineyard located at 45°56’22.5” N 12°18’05.2” E, in the hilly area of Ogliano (Northeast Italy). The vineyard is located at an altitude of 130 m a.s.l. on a South-West exposed hillside with a slope of approximately 30%. Before plantation, the land was subject to displacement and levelling to create terraces for vine cultivation and prevent the soil from erosion and sliding. The soil is sandy loam of glacial origin and classified as a Typical Udorthents loamy-skeletal, carbonatic, mesic (United States Department of Agriculture (USDA), 1999). The vineyard was planted in April 2019, using Cabernet sauvignon grafted onto Kober 5BB rootstock, and trained to a simple Guyot system with a spacing between plants of 2.10 x 1.0 m. No irrigation was applied, and vine management followed organic practices for disease and weed control. The under-row was managed performing mechanical weed control while the inter-row was kept covered by spontaneous grass. After the vineyard establishment, eight rows of approximately 80 vines were selected and randomly assigned to two fertilization treatments: compost amendment (Cmp) and fertilization with a mineral fertilizer (Fert). Compost was applied every year in spring in the under-row at a rate of 65 t ha^-1^. Mineral fertilization was performed every year in spring, about two-three weeks after budburst, by applying 80 kg/ha N, 50 kg/ha P_2_O_5_, 200 kg/ha K_2_O according to the recommended guidelines for the area (Fregoni, 2009).

The study-area is characterized by a mean precipitation of 700 mm and an average temperature of 17.8°C during the growing season (Tomasi et al., 2013). For each year of the study, the annual and seasonal (March–September) weather records for air temperature and rainfall, as measured by a nearby weather station (iMetos 3.3, Pessl Instruments, Weiz, Austria) are reported in **Table 1**.

**Table 1:** Annual and seasonal mean temperatures and total rainfall recorded in the study site during the three years of study.

	2019			2020			2021		
	MIN	MEAN	MAX	MIN	MEAN	MAX	MIN	MEAN	MAX
**T°C**_Jan - Dec_	6.7	15.0	26.0	5.8	15.0	26.2	4.8	14.4	26.2
**T°C**_1st Mar - 30th Sept_	10.1	19.3	31.4	9.0	19.3	30.6	8.9	18.8	30.9
**Rainfall ∑ mm**_Jan - Dec_	1769			1330			1358		
**Rainfall ∑ mm**_1st Mar - 30th Sept_	989			819			827		

### Compost characterization

The compost used in the experiment was produced every year by the farm from pruning residues, pomace and manure in a ratio of 30:30:40. Shredded materials were mixed and placed in piles subjected to a 3-months composting process and 6 months maturing phase. Every year, before amendment application, three samples of compost were randomly collected and sent to an external laboratory for analysis, following an official protocol of compost analysis methods, by Piedmont Region, environmental’s Assessor, Turin, 1998 (ARPA et al., 1998). Compost properties as average of the three years of experimentation is reported in **Table 2**.

**Table 2:** Main properties of the compost used in the experimental trial.

Compost properties	Mean ± SD
pH	7.73 ± 0.33
Dry matter (%)	56.3 ± 9.72
Moisture (%)	43.7 ± 9.69
Organic carbon (C) (% d.m.)	24.3 ± 5.37
Organic matter (% d.m.)	42.1 ± 9.12
Total nitrogen (N) (% d.m.)	1.59 ± 0.85
Carbon/nitrogen ratio (C/N)	17.1 ± 4.63
Total phosphorus (P) (% d.m.)	0.30 ± 0.17
Total potassium (K) (% d.m.)	0.76 ± 0.35
Salinity (mEq/100 g)	11.9 ± 5.49

d.m, dry matter.

The values represent the average of samples analyzed in three years (2019-2020-2021) ± SD.

### Soil sampling and analyses

Soil chemical and physical analyses were performed twice throughout the experiment: at the beginning of the study (April 2019), before the application of compost and fertilizer, and in the last year of the trial (April 2021). Three soil samples were randomly collected from the experimental site in 2019, and from the four rows of each treatment in 2021. Each sample was obtained mixing three sub-samples collected in the under-row, from 5 to 40 cm depth. Samples were then chemically analysed by an external laboratory according to methods foreseen by the Italian Ministry of Agriculture (Ministero dell’Agricoltura, 2004).

Soil microbial analysis was performed six times throughout the experimental trial. Every year samples were collected twice, in spring, at flowering, and in summer after harvest. At each sampling time, five soil samples were randomly collected for each treatment in the under-row, at a depth of 0 to 15 cm, as this layer is the most affected by soil management practices. Soil microbial biomass and functional (enzyme activity) analysis were performed six times throughout the experimental trial. Every year samples were collected twice, in spring, at flowering, and in summer after harvest. Five soil samples were randomly collected in the under-row, at depth of 0 to 15 cm, at each sampling time. Soil microbial biomass was determined as double-strand DNA (dsDNA) content as described in (Dal Cortivo et al., 2020)). Soil enzyme activity was determined as described in (Mattarozzi et al., 2020). The following enzyme activities, linked to different biogeochemical cycles were quantified: C-cycle: β-glucosidase, chitinase; N-cycle: leucine amino-peptidase; P-cycle: acid and alkaline phosphomonoesterase, phosphodiesterase, pyrophosphate phosphodiesterase, phytase; S-cycle: arylsulfatase.

### Soil metagenomic analysis

#### DNA extraction and amplicon pyrosequencing of bacterial and fungal communities

Total genomic DNA was extracted from 250 mg of fresh rhizosphere soil (from the last sampling on April 2021) using a PowerSoil Pro extraction kit (Qiagen, Germantown, MD, USA) according to manufacturing instructions. The bacteria-specific primers 341F-806R were used to amplify the V3-V4 region of the bacterial 16S rRNA gene, whilst the primer pair ITS3-ITS4 were used to amplify the ITS2 region of fungi. PCR amplicons were sequenced on an Illumina NovaSeq sequencing platform. Amplicon sequence variants (ASVs) were obtained from raw sequence data using DA.DA2 software. Briefly, the raw sequence reads were demultiplexed, adaptor-trimmed, quality-trimmed and denoised in order to remove sequences containing errors. Finally, the open source Vsearch software was used to blast clean tags to the database to detect the chimera and remove them (Rognes et al., 2016). The alignment of bacterial ASVs was conducted against SILVA 16S rRNA gene database using Wang’s classifier, while pairwise comparison with UNITE ITS database was performed for fungi ASVs.

#### Sequencing data analysis and calculation of biomarkers

Indices of taxonomic alpha diversity for bacteria and fungi were assessed using the R-packages “vegan” (for observed richness and Shannon) and “entropart” (for Chao1). Detection of treatment effects in alpha diversity was conducted with Kruskal-Wallis tests followed by Dunn’s *post-hoc* test (p < 0.05) with Bonferroni corrections using “pgirmess” R-package. Taxonomic beta diversity was assessed with Bray-Curtis dissimilarities using “vegan”. A Student’s t-test was then applied to evaluate possible eventual variation of microbial taxa between the treatments. In order to identify taxa characterizing the differences between the two treatments, the Linear Discriminant Analysis (LDA) Effect Size (LEfSe) (Segata et al., 2011) was implemented for high-dimensional biomarker discovery and explanation. ASVs with a LDA log score > 3.0 were used for interpretation. Differences in community composition between Fert and Cmp samples were evaluated by the Principal Coordinate Analysis (PCoA), Non-Metric Multi-Dimensional Scaling (NMDS) and Principal Component Analysis (PCA) run with the Adonis function in the R package “vegan”.

### Leaf chlorophyll content

Leaf chlorophyll content was measured on forty randomly selected plants from the four rows of each treatment in early June and early August at the flowering and veraison stages according to the BBCH scale (Lorenz et al., 1995). Fully expanded leaves inserted opposite to the basal bunches on main shoots were randomly chosen in number of one per plant, and readings were recorded by a portable chlorophyll meter (SPAD-502 Plus, Konica-Minolta, Osaka, Japan). The instrument calculated a numerical SPAD value, which is proportional to the amount of chlorophyll in leaf tissues, considered as an indicator of the nitrogen nutritional status of plant.

### Vegetative growth, yield and quality parameters

Vegetative growth was measured every year at the beginning of August by digital image analysis. Measurements were taken on twenty randomly chosen plants per treatment. For each selected plant, a white panel provided with dimensional markers was positioned in the background. Image acquisition was performed using a digital camera placed on a tripod 1.5 m far from the row, framing the canopy with an angle of 90°. Images were analyzed using the open-source software ImageJ (National Institutes of Health, USA, 1997) as described by (Schneider et al., 2012). The value of green area represented by shoots and leaves for each plant was then calculated and considered as an indicator of the plant vegetative growth.

In 2021, the third year after vineyard planting and the actual first productive one, yield and quality parameters, total soluble solids, titratable acidity and pH, were measured at harvest on twenty-four vines per treatment. Yield per vine and average cluster weight were recorded using a hanging scale (CH, Kern, Germany). Three samples of grapes of approximately 1 Kg were randomly collected from the harvested plants of each treatment and immediately brought in laboratory for chemical analysis. Soluble solids, titratable acidity, pH, and the content in tartaric and malic acids were then determined in the must extracted from each sample. Soluble solids content (°Brix) was determined by a digital refractometer (ATAGO PR-32, Thermo Fisher Scientific, Waltham, USA) at 20°C; titratable acidity (expressed as g L^-1^ of tartaric acid equivalents) was determined using an automatic titrator (Crison Micro TT 2022; Crison Instruments, Barcelona, Spain) by titration with NaOH 1 N; pH was measured by the electrode (Flush Trode sensor, Hamilton Company, USA). Malic and tartaric acid contents (g L^-1^) were determined chromatographically using the RP-HPLC method (Agilent 1220 Infinity LC, Agilent Technologies, Santa Clara, CA, USA). The procedure consisted in a 40-fold dilution of must samples followed by a filtration through 0.2 mm sterile cellulose acetate membrane (Whatman 6901-2502) and the preparation of an injection volume of 20 µL for each sample. Separations were carried out under isocratic conditions at 36°C using a H_3_PO_4_ 0.01 M solution as mobile phase with a 0.6 mL min^-1^ flow rate. Organic acids were detected at 210 nm wavelength as described by Kordiš-Krapež et al. (2001).

Yeast Assimilable Nitrogen (YAN) in must was quantified by a spectrophotometer (UV Mini-1240, Shimadzu Europe, Duisburg, Germany) according to (Nicolini et al., 2004).

### Statistical analyses

The significance of differences between Cmp and Fert treatments for soil properties, microbial biomass, enzyme activities and for plant agronomic parameters was assessed by Student’s t-test. Prior to the t-tests, all experimental data were submitted to Shapiro-Wilk test for normality. The tests, as well as the descriptive statistics, were performed using the software Statistica 8.0 (StatSoft Inc., Tulsa, OK, USA).

Taxonomic alpha- and beta-diversity for bacteria and fungi in the soil were determined through dedicated R packages as reported in detail in “Soil metagenomic analysis” section.

## Results

### Soil properties

As shown in **Table 3**, compost amendment (Cmp) improved significantly the main physicochemical properties of the soil at the third year of application (2021). Comparing Comp and Fert treatments on the last year of study, the most relevant difference concerned the amount of soil organic matter (SOM) with values (%) equal to 10.2 and 3.8 for soil treated with compost and fertilizer, respectively. Compared to the pre-treatment, a high availability of all macronutrients was found in the soil of both treatments in 2021. Despite the growth in both treatments, the positive effect of compost in the soil is evident for the main nutrients. Total N increased significantly (+227%). The greatest increase was in assimilable P with a 340% net increase compared to Fert, and also in exchangeable K with a 366% increase.The C/N ratio was slightly higher for Cmp (9.9 vs 8.6). With regard to the temporal change, comparison of the pre-treatment analysis (2019) with those performed at the third year of study showed a general increase in soil organic matter, organic carbon and nutrients content, more marked in Cmp than in the Fert treatment (**Table 3**). Differently from the amended soil, the fertilized one did not show a significant increase in exchangeable calcium and in the C/N ratio compared to 2019. Furthermore, the application of compost did not increase the level of exchangeable sodium in soil, whereas a slightly reduction, although not significant, was observed in fertilized soil (**Table 3**).

**Table 3:** Main physicochemical properties of the soils at the first year at the study (2019) and at the third year (2021), for plots treated with compost (Cmp) and with a fertilizer (Fert).

Soil properties	2019	2021	
	Pre-treatments	Cmp	Fert
Organic matter (%)	2.4 ± 0.42	10.2 ± 0.64a*	3.8 ± 0.41b*
Organic carbon (%)	1.4 ± 0.24	5.9 ± 0.37a*	2.2 ± 0.24b*
Total nitrogen (N) (%_0_)	1.6 ± 0.23	5.90 ± 0.34a*	2.6 ± 0.27b*
Available phosphorus (P_2_O_5_) (ppm)	37.0 ± 9.89	431 ± 91.1a*	127 ± 9.29b*
Exchangeable potassium (K_2_O) (ppm)	128 ± 14.1	1111 ± 222a*	303 ± 29.1b*
Exchangeable sodium (Na) (ppm)	34.5 ± 4.95	33.0 ± 2.0a	27.3 ± 1.15b
Exchangeable calcium (Ca) (ppm)	2594 ± 289	3665 ± 62.4a*	3029 ± 82b
Exchangeable magnesium (Mg) (ppm)	160 ± 19.8	570 ± 73.5a*	179 ± 4.04b*
Carbon/nitrogen ratio (C/N)	8.8 ± 0.23	9.9 ± 0.06a*	8.6 ± 0.29b
pH	7.88 ± 0.04	8.06 ± 0.05	8.24 ± 0.12*
	**Soil Physical Characterization**		
Sand %	27		
Clay %	25		
Silt %	48		

The values represent the mean of three replicates ± SD (standard deviation). Significant differences between pre-treatment values and each treatment in 2021 were determined by Student’s t-test (p<0.01) and are indicated with asterisks. Significant differences between Cmp and Fert for the year 2021 were determined by Student’s t-test (p<0.01) and are indicated with letters.

### Soil microbial activity

**Figure 1:**
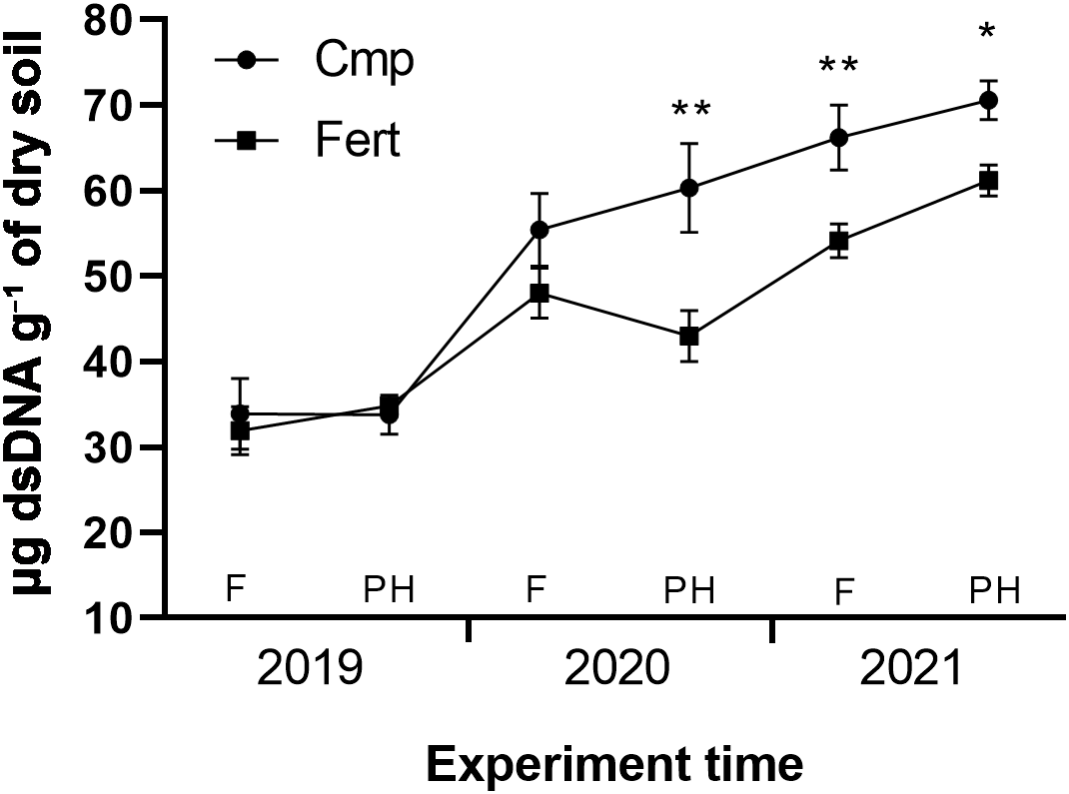
Soil microbial biomass evolution trend throughout the study. Within each year soil samples were analyzed at flowering (F) and at post-harvest (PH) phenological stages. Data are means of five replicates ± SE. Unpaired Student’s t-test was performed between Cmp and Fert for each sampling time. Asterisks indicate statistically significant differences (p<0.05 *, p<0.01 **).

**Figure 1** shows the microbial biomass index, expressed as micrograms of dsDNA g^-1^ dry soil, over the three years of the experimentation. In the first year (2019) no significant differences between the two treatments were observed, with values ranging between 32-35 μg dsDNA g^-^¹ of dry soil. In the second year (2020) the microbial biomass increased in both treatments, with a mean annual increase of +63% and +48% for Cmp and Fert, respectively, compared to 2019 values. A significant difference between Cmp and Fert was observed in post-harvest. The increasing trend of microbial biomass was confirmed also in 2021 (mean annual increase of +100% for Cmp and +72% for Fert, compared to 2019 values), with significantly higher values for Cmp at both sampling times (flowering and post-harvest).

**Table 4** shows the different responses of the enzymatic activities to the treatments over the three years of the study. In 2019, the application of compost had not significant influence on the enzyme activity, although vines treated with fertilizer tent to show slightly higher activities values. In the second year of the study (2020), β-glucosidase, chitinase and leucine-aminopeptidase were still not affected by the application of compost, as Fert and Cmp showed similar values for these enzymes. By contrast, changes for the enzymes involved in the phosphorus cycle were observed, with a relevant increase in Cmp: the two phosphomonoesterases, acid and alkaline, showed 24% and 34% higher values, respectively, for Cmp compared to Fert. A similar increase was observed also for pyrophosphate phosphodiesterase and phosphodiesterase (**Table 4**). Arylsulfatase was also found to be influenced by the compost, with a higher value for Cmp than Fert (22 vs 15 nanomoles of 4-MUF h^-1^ g^-^¹ dry soil). In the third year (2021), all the enzymatic activities showed a significant positive response to the compost treatment, except for β-glucosidase. Chitinase, an enzyme related to the carbon cycle, showed a significantly higher activity in Cmp than in Fert (16.5 vs 11.4 nanomoles of 4-MUF h^-1^ g^-^¹ dry soil). The activity of the enzyme leucyl-aminopeptidase, which takes part to the nitrogen cycle, was also positively influenced by the compost (93.2 vs 79 nanomoles of 4-MUF h^-1^ g^-^¹ dry soil in Cmp and Fert, respectively). In line with the 2020 trend, all the phosphorus cycle enzymes showed higher values in Cmp treatment than in Fert. In detail, acid and alkaline phosphatase showed an increased activity in Cmp. pyrophosphate-phosphodiesterase and phosphodiesterase activities were 20% and 32% higher than the Fert treatment. Finally, arylsulfatase, involved in the sulphur cycle, confirmed the 2020 results, showing higher activity in the amended soil, with value equal to 30.5 and 23.7 nanomoles of 4-MUF h^-1^ g^-^¹ dry soil respectively for Cmp and Fert.

**Table 4:** Enzymatic activities measured in soils during the three years of experimentation.

	2019		2020		2021	
	Cmp	Fert	Cmp	Fert	Cmp	Fert
β-Glucosidase	4.1	6.8	10	10	8.1	7.4
Chitinase	3.7	6.3	12	9	16.5 **	11.4
Leucyl aminopeptidase	22.3	34.7	261	226	93.2 *	79.0
Acid phosphatase	35.4	67.7	46 *	37	75.4 ***	61.9
Alkaline phosphatase	133.8	229.2	1332 **	993	730.5 **	541.4
Pyrophosphate - Phosphodiesterase	13.6	27.7	11 **	8	20.3 ***	16.9
Phosphodiesterase	31.7	54.3	165 ***	111	102.3 ***	77.4
Arylsulfatase	25.4	45.7	22 **	15	30.5 ***	23.7

Enzyme activity is expressed in nmol of Hymecromone g^-1^ soil h^-1^. Leucyl aminopeptidase activity is expressed as C_10_H_9_NO_2_ nmol g^-1^ soil h^-1^. Data are means of five replicates ± SD. Statistical analysis was performed using unpaired Student’s t-test to compare treatments within each year. Asterisks indicate significant differences (p<0.05 *, p<0.01 **, p<0.001 ***).

### Soil microbial communities

#### Microbial community associated with rhizosphere soil of grapevine: bacteria

**Figure 2:**
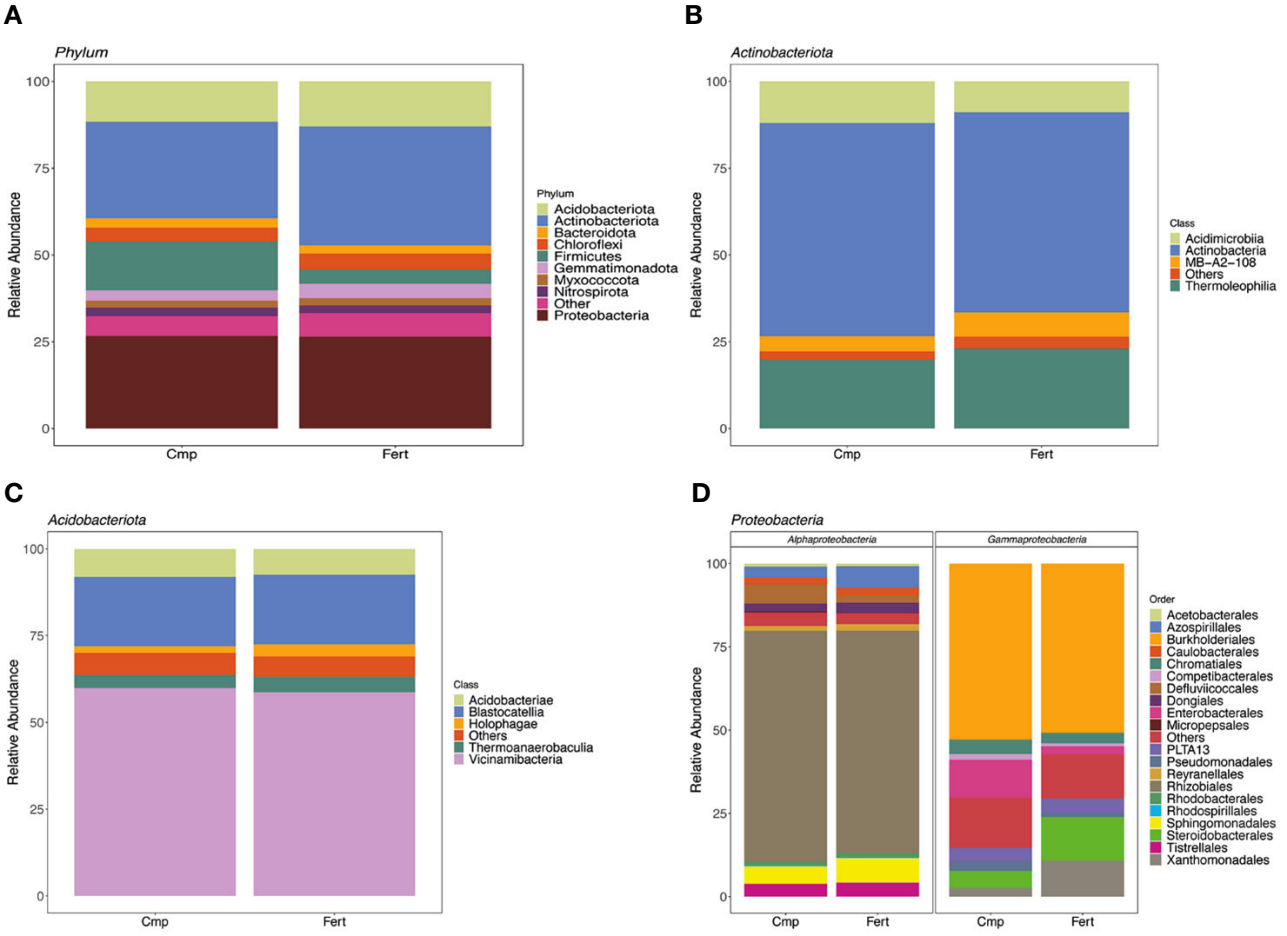
Taxonomic analysis of microbial community. **(A)** The composition and relative abundance of major bacterial taxa at phylum level in rhizosphere soil of grapevine plants either fertilized (Fert) or treated with compost (Cmp). **(B)** Composition and relative abundance of major bacterial classes from the phyla of Actinobacteriota. **(C)** Composition and relative abundance of major bacterial classes from the phyla of Acidobacteriota. **(D)** Composition and relative abundance of major bacterial order from the classes Alpha- and Gammaproteobacteria. Each bar represents the average value of four replicates in each sample group.

After assembly and quality filtering, a total of 166,381 high quality bacterial gene sequences (average of 20,798 ± 2,586 reads per sample) was obtained (**Supplementary Table 2**). At phylum level, independently from the treatment applied, the rhizosphere microbial community associated with grapevine plants was dominated by Actinobacteriota (31.0%± 6.4), followed by Proteobacteria (26.6% ± 3.7), Acidobacteriota (12.3% ± 2.1), Firmicutes (9.1% ± 6.9), Chloroflexi (4.3% ± 0.5), Gemmatimonodota (3.6% ± 0.8), Bacteroidota (2.6% ± 0.6), Nitrospirota (2.4% ± 0.7) and Myxococcota (2.1% ± 0.4). Interestingly, when comparing the relative abundance of the bacterial phyla detected, Actinobacteriota and Gemmatimonodota were selectively enriched in the rhizosphere of fertilized plants, whilst Firmicutes were strongly enriched (by about three times) in the rhizosphere of plants treated with compost (**Figure 2A**, **Supplementary Table 3**).

**Figure 3:**
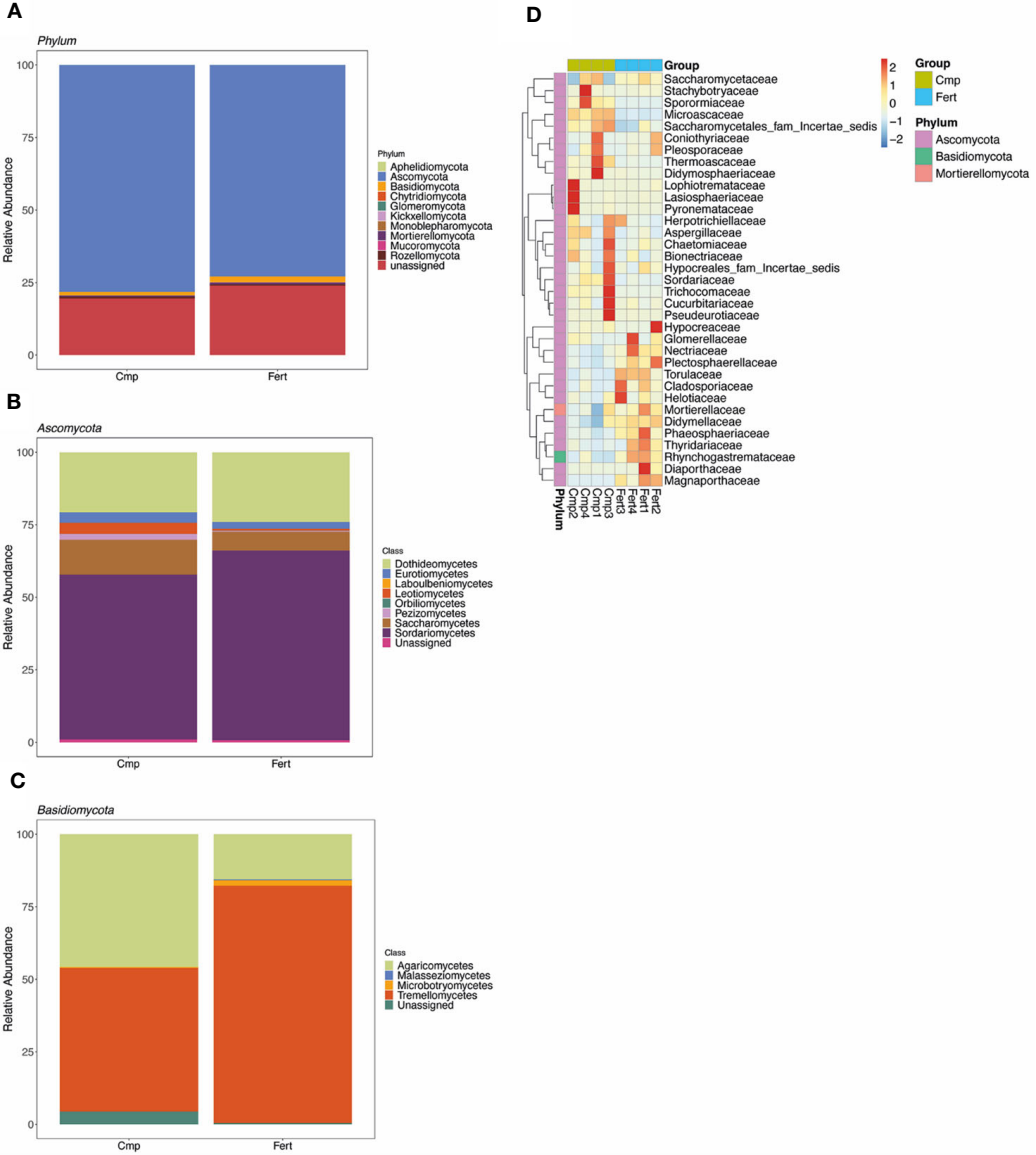
Taxonomic analysis of fungal community. **(A)** The composition and relative abundance of major fungal taxa at phylum level in rhizosphere soil of grapevine plants either fertilized (Fert) or treated with compost (Cmp). **(B)** Composition and relative abundance of major fungal classes from the phyla of Ascomycota. **(C)** Composition and relative abundance of major fungal classes from the phyla of Basidiomycota. Each bar represents the average value of four replicates in each sample group. **(D)** Cluster map of species abundance at different taxonomic levels. The x-axis represents the sample name, and the y-axis represents the function annotation. The cluster tree on the left side of the figure is the species cluster tree; the corresponding value of the heatmap is the Z value of taxonomic relative abundance after standardization, that is, the Z value of a sample in a certain species is the differences between the relative abundance of the sample in the species and the average relative abundance of all samples in the species divided by the standard deviation of all samples in the species.

In the case of Actinobacteriota, the phylum was dominated by the class of Actinobacteria (59.5% ± 5.9), followed by Thermoleophilia (21.4% ± 4.1) and Acidimicrobiia (10.4% ± 2.5); in the specific case of Acidimicrobiia a slight enhancement was observed in Cmp samples compared to Fert (**Figure 2B**, **Supplementary Table 3**). For the phylum Acidobacteriota (**Figure 2C**; **Supplementary Table 4**), we observed a predominance of Vicinamibacteria (59.3% ± 2.4 and Blastocatellia (20.0% ± 3.2). With regard to Proteobacteria, we investigated the composition at class level, and we found that it was mainly composed by Alpha- and Gammaproteobacteria (**Figure 3D**; **Supplementary Table 5**). The Alphaproteobacteria class was mainly represented by members of the order Rhizobiales (68.1% ± 4.1), Azospirillales (4.9% ± 1.9), and Sphingomonadales (6.3% ± 1.2), whilst, within Gammaproteobacteria, we observed Burkholderiales (51.8% ± 3.5), Steroidobacterales (9.1% ± 4.9), Enterobacterales (7.0% ± 9.9) and Xanthomonadales (6.7% ± 7.0). Among these, Enterobacterales were well represented in Cmp samples, whereas Azospirillales and Xanthomonadales were depleted (**Figure 2D**; **Supplementary Table 4**).

#### Microbial community associated with rhizosphere soil of grapevine: fungi

After quality trimming and the removal of chimeras a total of 270,262 high-quality fungal sequences (an average of 33,783 ± 3040 sequences per sample) was obtained (**Supplementary Table 2**). At phylum level, the fungal population associated with the rhizosphere analysed was dominated by Ascomycota (75.5% ± 7.6), followed by Basidiomycota (1.6% ± 0.7) (**Figure 3A**; **Supplementary Table 6**). Nevertheless, at this taxonomic level we could not highlight any significant difference between the treatments imposed (**Figure 3A**; **Supplementary Table 7**). Within the phylum Ascomycota, the most abundant class was represented by Sardariomycetes (60.2% ± 13.2), followed by Dothideomycetes (22.2% ± 11.8), Saccharomycetes (9.6% ± 4.4) and Eurotiomycetes (3.3% ± 1.9), whilst the phylum Basidiomycota resulted mainly dominated by Tremellomycetes (65.6% ± 25.4) and Agaricomycetes (30.7% ± 25.3) (**Figures 3B, C**; **Supplementary Table 7**). However, when the relative occurrences of the different classes were compared between Cmp and Fert samples, any significant difference could be highlighted.

On the other hand, according to the species annotations and abundance information of all samples at the genus level, by selecting the top 35 genera in abundance, and clustering them considering the species and sample as variables, the heatmap obtained clearly showed a different trend in the fungal population depending on the treatment imposed (**Figure 3D**), particularly showing a shift in the share of genera included in the Ascomycota phylum. For instance, the genera *Diutina*, *Mycochlamys*, *Aspergillus*, *Paracremonium*, *Clonostachys*, and *Thermoascus* were more dominant in Cmp treatment, whilst *Paraphoma*, *Torula*, *Cladosporium*, *Fusarium*, *Plectosphaerella*, *Papiliotrema*, *Mortierella*, and *Mycoleptodiscus* dominated in Fert samples.

#### Alpha− and beta diversity of bacterial and fungal communities associated with grapevine plants

Bacterial and fungal α-diversity in the samples of each treatment was evaluated by applying ASV-based analysis methods and they failed to highlight any significant difference in alpha-diversity parameters of the tested communities (**Supplementary Figure 1**, bacteria: observed *p*-value = 0.0830, Chao1 *p*-value = 0.0833 and Shannon *p*-value = 0.0433; fungi: observed *p*-value = 0.8845, Chao1 *p*-value = 1.0 and Shannon *p*-value = 0.7728).

**Figure 4:**
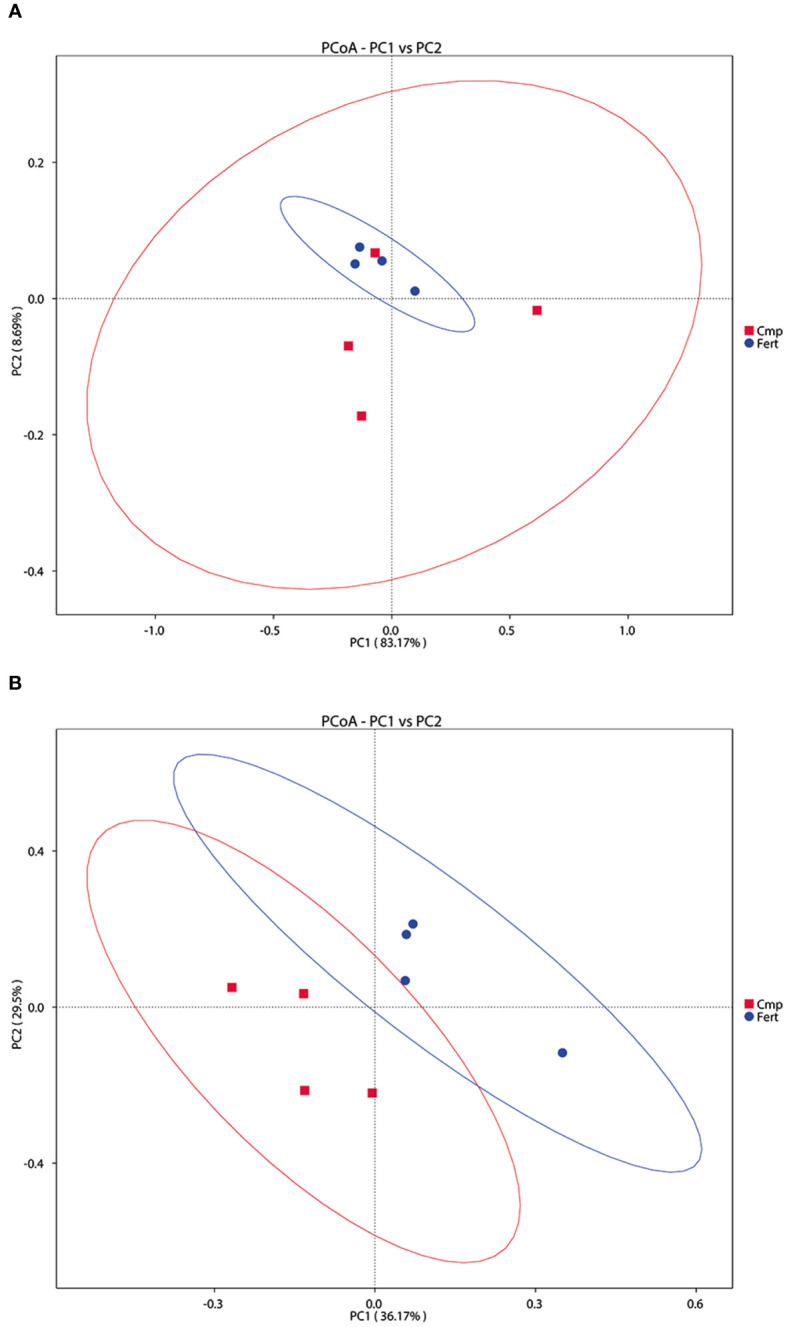
Beta diversity analysis to estimate the dissimilarity and similarity of microbial communities’ composition among different samples. **(A)** Principal coordinated analysis (PCoA) derived from dissimilarity matrix of weighted UniFrac distance depicting the diversity of bacterial community. **(B)** Principal coordinated analysis (PCoA) derived from dissimilarity matrix of weighted UniFrac distance depicting the diversity of fungal community.

**Figure 5:**
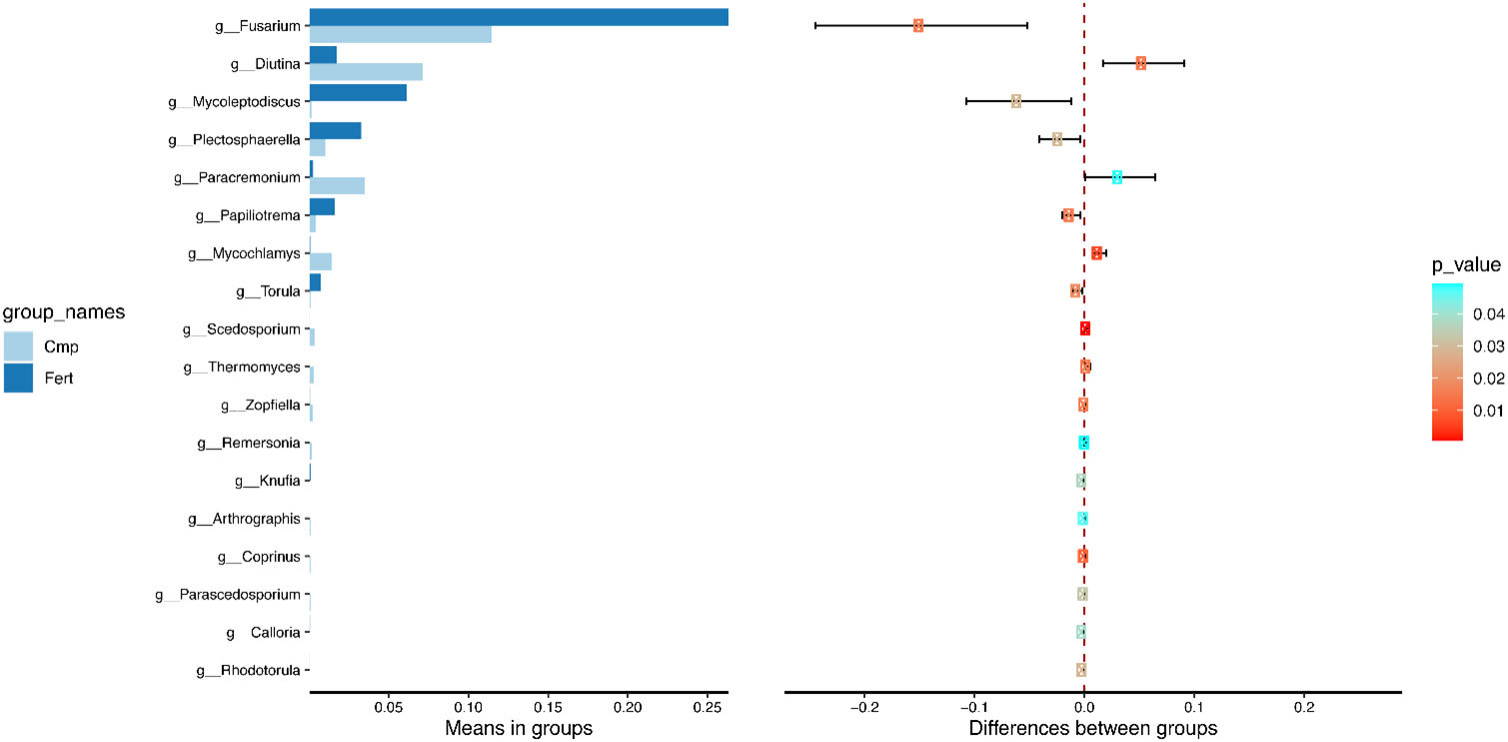
T-test analysis of species differences between treatments. The statistical test was performed to determine species with significant variation between groups (p value < 0.05) at genus level. The left figure shows the species abundance differences between groups, and each bar in the figure represents the average abundance of the species in different groups; the right figure shows the confidence of inter-group differences. The leftmost point of each circle in the figure represents the lower limit of 95% confidence interval, and the rightmost point of the circle represents the upper limit of 95% confidence interval. The center of the circle represents the differences of the mean value, and the color of the circle represents the P value of differences significance test between groups of the corresponding species.

On the other hand, the β-diversity of the bacterial and fungal communities in the rhizosphere is summarized in **Figure 4**. The PCoA analysis of the bacterial communities did not show a clear clustering according with the different treatment (ADONIS *p*-value = 0.679), albeit Fert displayed a tendency of grouping together respect to Cmp. These observations were further confirmed also by PCA and NMDS analyses (**Supplementary Figures 2A, B**). However, in the case of fungal communities, the PCoA analysis revealed that samples from the two treatments - formed clusters separated along the first axis (ADONIS *p*-value = 0.029); these observations were also confirmed by PCA and NMDS analyses (**Figure 5**, **Supplementary Figure 2**).

To further explore the differences observed by the fungal β-diversity analysis, the Student’s t-test showed that Cmp samples featured a significant decrease in *Fusarium*, *Mycoleptodiscus*, *Plectosphearella* and *Papiliotrema*, whereas *Diutina* and *Paracremonium* increased significantly (**Figure 5**).

#### Identification of biomarkers

**Figure 6:**
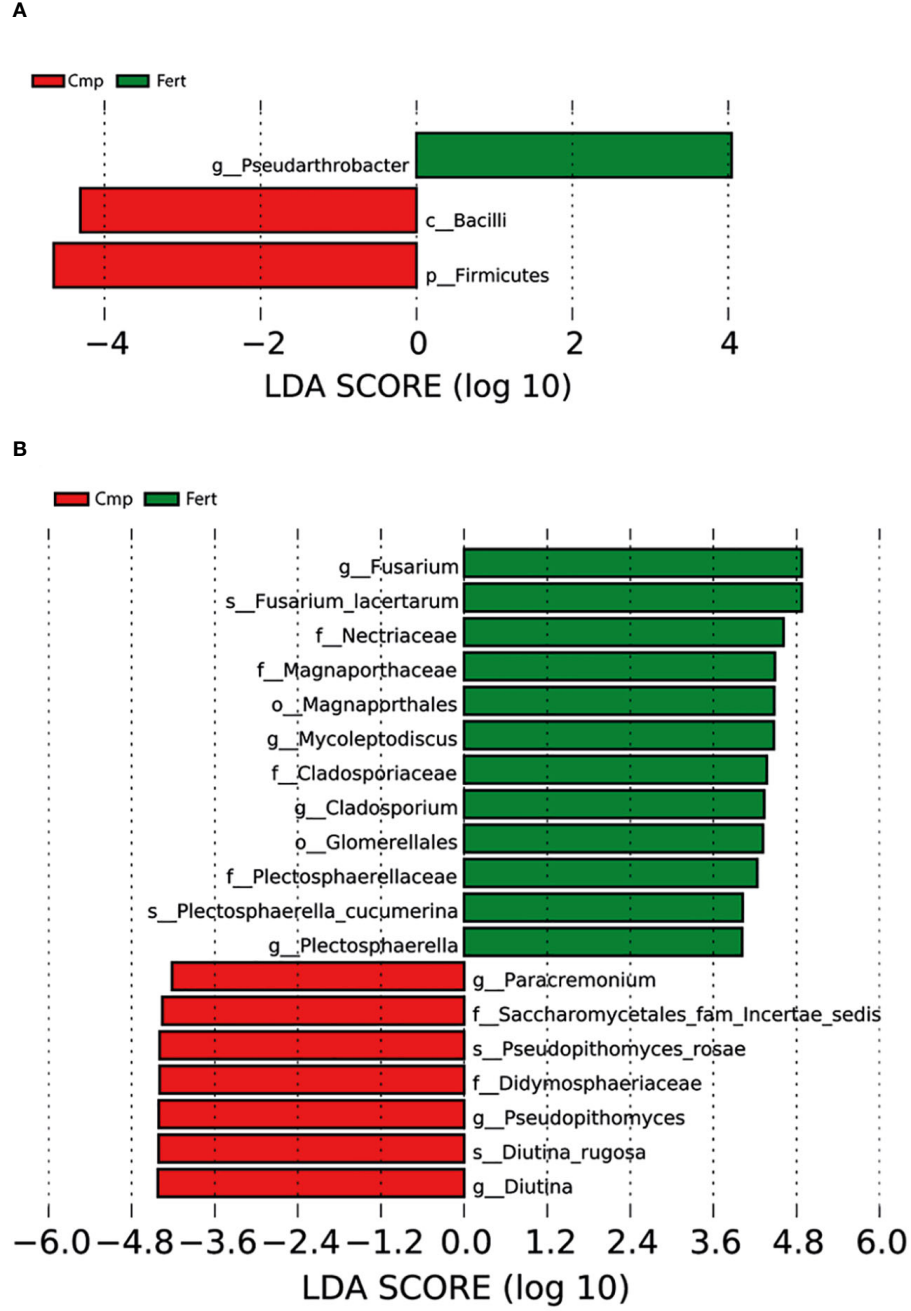
Linear discriminant analysis effect size (LEfSe) (log10 LDA score) of **(A)** bacterial and **(B)** fungal ASV detected as indicator representants for Fert (green) and Comp (red) samples from the rhizosphere of grapevine. Bacterial and fungal ASV are classified at the highest resolvable taxonomic level. The x axis scaling is represented by the log10 of LDA score, which is determined through a Linear Discriminant Analysis (LDA) to estimate the biomarker relevance, as previously demonstrated by Segata et al., 2011.

The sequencing data was further subjected to LEfSe analysis to discover high-dimensional biomarkers that could potentially discriminate the composition and the functioning of the bacterial communities associated with the two treatments imposed. The LEfSe analysis highlighted that Cmp samples were characterized by ASVs belonging to the phylum of Firmicutes, as also highlighted in **Figure 3** and **Supplementary Table 2**, and to the class of Bacilli (**Figure 6A**). On the other hand, Fert samples resulted characterized by a selective enrichment in bacterial species belonging to the genus *Pseudoarthrobacter* in the phylum of Actinomycetota, which were not discovered in the above-mentioned analysis of the community composition and structure (**Figure 6A**). In case of fungi, the LEfSe analysis highlighted that Cmp samples were characterized by ASV belonging to the family of Saccharomycetales and Didymosphaeriaceae, to the genus of *Paracremonium*, *Pseodopithomyces* and *Diutina* and to the species of *Pseudopithomyces rosae* and *Diutina rugosa* (**Figure 6B**). Conversely, Fert samples resulted characterized by a selective enrichment in fungal ASV belonging to the order of Magnaporthales and Glomerellales, to the family of Nectriaceae, Magnaporthaceae, Cladosporiaceae and Plectosphaerellaceae, to the genus of *Fusarium*, *Mycoleptodiscus* and *Plectosphaerella* and to the species of *Fusarium lacertarum* and *Plectosphaerella cucumerina* (**Figure 6B**).

### Leaf chlorophyll content and plant agronomic responses

**Figure 7:**
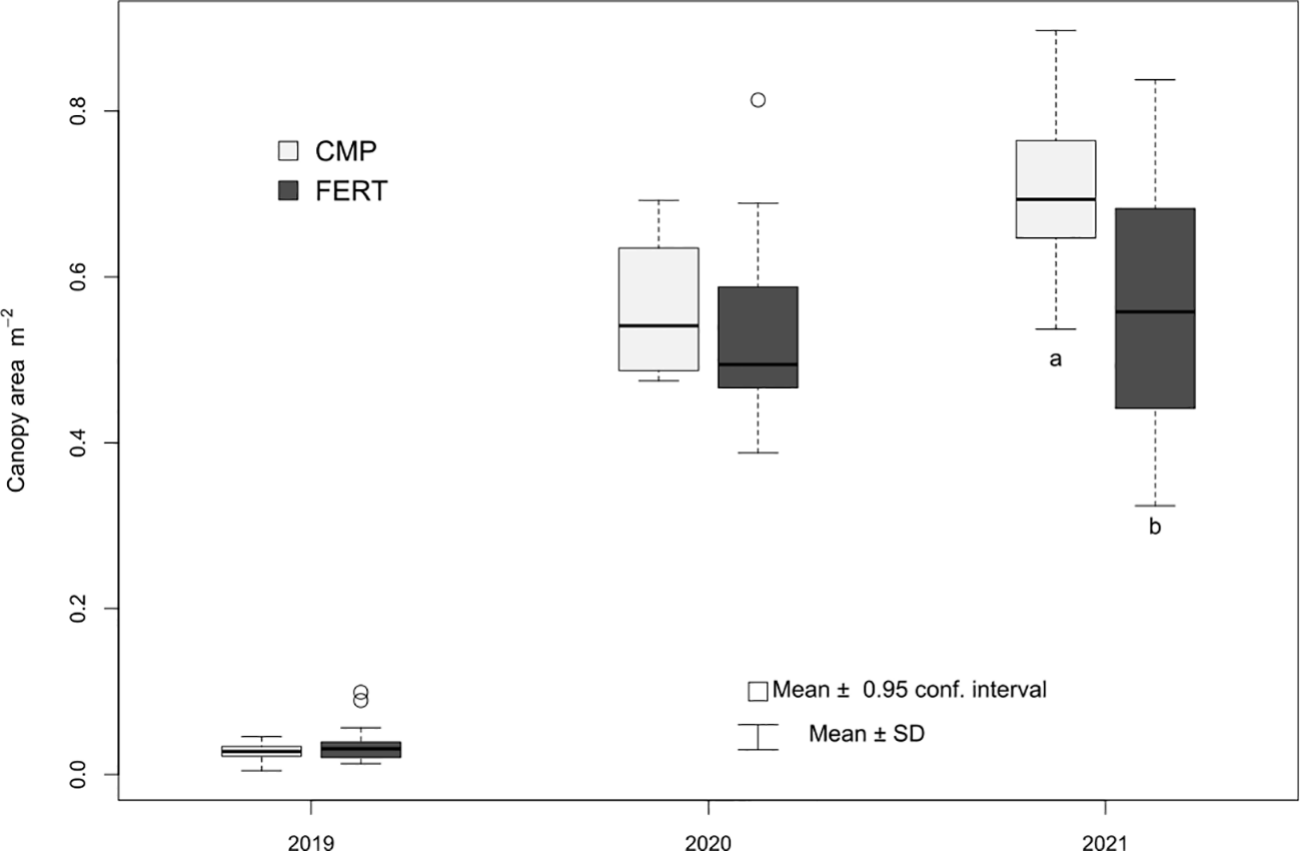
Canopy area (m2) calculated at veraison for grapevine plants treated with compost (Cmp) and with a fertilizer (Fert) as control. Different letters indicate significant differences between treatments for within year (Student’s t-test; p<0.05.

The SPAD index, related to the content of leaf chlorophyll and nitrogen nutritional status of plants, did not show significantly different values between Cmp and Fert in 2019 and 2020, neither at flowering nor at veraison. More evident differences were observed in 2021 at both phenological stages, particularly at flowering, when the compost-treated grapevines showed significantly higher SPAD values than the fertilized ones (**Table 5**). In agreement with the nitrogen nutritional status of plants, the vegetative growth was not affected by the treatments in the first and second year of study, while a positive effect was observed in the third year (2021), as the vegetative growth measured at veraison showed significant higher values for the vines treated with compost (0.70 vs 0.56 m^2^; p<0.01) (**Figure 7**).

**Table 5:** SPAD index values ± SD measured at flowering and veraison for grapevine plants treated with compost (Cmp) and with a fertilizer (Fert).

Year	Phenological phase	Treatment	
		Cmp	Fert
**2019**	Flowering	24.7 ± 3.75	23.0 ± 2.88
	Veraison	30.8 ± 3.03	30.8 ± 2.73
**2020**	Flowering	31.2 ± 2.22	31.6 ± 3.21
	Veraison	39.6 ± 3.85	38.5 ± 3.89
**2021**	Flowering	37.8 ± 1.95a	36.1 ± 2.63b
	Veraison	38.3 ± 4.14	36.6 ± 3.35

Different letters indicate significant differences between treatments within each phenological stage (Student’s t-test; p<0.05).

**Table 6** shows the yield and the quality parameters of grapevines recorded at harvest in 2021. The application of compost did not significantly affect the production per plant (0.91 vs 0.72 Kg for Cmp and Fert, respectively). With respect to the grape quality, the use of compost did not affect the sugar content (°Brix), but a significant increase in titratable acidity (5.9 vs 5.1 g L^-1^) and malic acid content (2.7 vs 2.1 g L^-1^) could be observed in Cmp treatment. Grapes from plants treated with compost showed also higher yeast assimilable nitrogen values compared to those from Fert treatment (75.6 vs 59.0 mg L^-1^) although differences were not statistically significant.

**Table 6:** Production and qualitative parameters of grapevine plants treated with compost (Cmp) and with a fertilizer (Fert) in the first year of production (2021).

parameters	Cmp	Fert
	Mean ± SD	
Yield (Kg/plant)	0.91 ± 0.26	0.72 ± 0.25
Bunch mean weight (g)	91.0 ± 6.8b	102.0 ± 5.8a
Sugar content (°Brix)	22.1 ± 0.6	22.1 ± 1.1
Titratable acidity (g/l)	5.9 ± 0.2a	5.1 ± 0.3b
pH	3.41 ± 0.31b	3.51 ± 0.01a
Tartaric acid (g/l)	5.6 ± 0.1	5.7 ± 0.2
Malic acid (g/l)	2.7 ± 0.2a	2.1 ± 0.3b
Yeast assimilable nitrogen (mg/l)	75.6 ± 20.6	59.0 ± 6.1

Different letters indicate significant differences between treatments (Student’s t-test; p<0.05).

## Discussion

In viticulture, more than in other agronomic fields, the concept of terroir plays a significant role. A wine is the expression of a defined geographic area, and the chemical, physical and biological characteristics of the soil are reflected in a unique and specific wine quality profile, not reproducible elsewhere. Vineyard planting, when involves slope reshaping, can strongly impacts soil. Agricultural practices, such as application of mineral or organic fertilizers, can also change the physical and chemical properties of the soil, with an impact on the quality of the grapes that grow from it.

Previous literature agrees that the benefits derived from the use of compost in vineyard can be primarily attributed to the increase in SOM (Bustamante et al., 2011; Calleja-Cervantes et al., 2015; Gaiotti et al., 2017; Mondini et al., 2018). In agreement to these studies, in our trial SOM increased significantly in response to compost application compared to the soil treated with fertilizers. As a result of the high rates of compost applied (650 q/ha), after 3 years of amendment SOM content in Cmp reached values more than double those of Fert treatment. It is indeed known that the amount of SOM increases linearly with the amount of compost applied (Mondini et al., 2018).

As a major reservoir of mineral elements, (Gerke, 2022), high SOM in soil is expected to increase nutrient availability in the soil thus promoting plant growth (Reeves, 1997). Total soil nitrogen is a key indicator of nitrogen availability in the soil and is closely related to soil productivity (Al-Kaisi et al., 2005; Liu et al., 2022). Nitrogen plays a key role in growth and development of grapevine being essential to build compounds such as proteins, enzymes, amino acids, nucleic acids, and pigments including chlorophyll and anthocyanins of berries (Verdenal et al., 2021). Following the application of compost, our analyses revealed an increase in the total N content in the soil. After 3 years of compost application total N content resulted approximately 2-fold higher in Cmp than in Fert treatment. Similar effects in vineyard soils were observed by other authors, after five (Gaiotti et al., 2017) and six years (Pinamonti, 1998) of compost application. Carbon-to-nitrogen ratio (C/N) is a reliable indicator of soil quality and represents a useful index to explain different turnover rates for residue decomposition and N cycling in soils (Li et al., 2016; Akratos et al., 2017). In the present study, the soil treated with compost showed an increase in C/N ratio. This trend implies a slower mineralization of organic matter, with a gradual release of available N for plant uptake in Cmp treatment (Brust, 2019).

Compost treatment induced a significant increase of available P, with values three- fold higher in Cmp than in Fert. Soil P has been shown to typically increase after compost application due to inhibition of P sorption sites by organic acid anions, as well as to the additional mineral and organic P applied (Hue, 1992; Korboulewsky et al., 2002; Hunt et al., 2007; Wilson et al., 2016). Further, the consistent application of mixed compost, as in our study, results in an increase in soil organic matter, which is the main source of organic phosphate compounds for soil microorganisms. The greater availability of assimilable P in the Cmp treatment compared to Fert is also related to this mineralisation by fungi and bacteria through specific enzymes such as phosphoromonesterase and, to a lesser extent, phosphodiesterase and pyrophosphatase (Billah et al., 2019).

As for N and P, in our study exchangeable K in soil increased significantly with the use of compost. The role of K in grapevine nutrition is crucial. Potassium is the major cation in grape juice and has a key role in pH regulation (Mpelasoka et al., 2003; Rogiers et al., 2006). Increases in soil K have been observed in a wide range of studies, using compost form different sources, and applied at different rates (Pinamonti, 1998; Larchevêque et al., 2006; Morlat and Chaussod, 2008; Bustamante et al., 2011; Chan and Fahey, 2011; Calleja-Cervantes et al., 2015). Compost is usually rich in potassium, and when available, vines tend to uptake excess of K and accumulate it in the berry. This can negatively affect wine pH, reducing wine stability and worsening grape colour and aromas (Mpelasoka et al., 2003). The amount of other exchangeable cations, such as Mg, involved in must fermentative metabolism (Walker et al., 1990), and Ca, a key component of cell walls (Wang et al., 2022), were also significantly increased by compost application, confirming the findings from previous studies that showed a correlation between the availability of these nutrients and the amount of organic matter in the soil (Zink Ret and Allen, 1998; Ramos et al., 2018).

Microbial biomass and activity are widely recognized as indicators of soil health and have been increasingly used to investigate changes due to agronomic practices or environmental disturbance (Kandeler, 2015). While soil operations such as land levelling facilitate vine cultivation on steep slopes, they tend to alter the soil profile, both physically (Rodrigo-Comino et al., 2017), and biologically (Mondini et al., 2018), increasing the risk of degradation and fertility loss.

Addition of organic fertilizers has reported to increase soil microbial biomass through the supply of carbon organic compounds to the soil microbial communities (Diacono and Montemurro, 2011). Interestingly, in our study microbial biomass showed a progressive increase over the three years of the experiment in both treatments. This trend suggests that, regardless the application of compost, microbial biomass might have recovered in both treatments after a disturbance caused by land levelling. In support of this, Mondini et al. (2018) reported that land levelling significantly affected the microbiota in vineyard soils, with disturbed soils displaying lower values of microbial biomass compared to undisturbed ones.

Comparing the microbial biomass in soils treated with compost or fertilizer, we did not observe differences in the first year (2019), but a significant variation was observed from the second year onwards, with higher values for Cmp. This difference in microbial biomass growth can be attributed to increased SOM levels in soil following the application of compost, as confirmed by the physio-chemical analysis of Cmp soil samples. According to results of previous studies (Fließbach et al., 2000; Khan et al., 2016), the organic matter supplied by the compost stimulates the soil community by providing a nutrient-rich substrate and creating a favourable environment for microbial development. In our study microbial biomass showed a slower response to compost application than that observed in other trials. (Lazcano et al., 2013), for instance, observed significant effects of compost amendments on soil microbial biomass after only 3 months from compost application. However, variation in the timing and magnitude of microbial biomass response might be tied to the initial physical and biological soil conditions, as well as to the type and rate of compost applied. In fact, the compost applied in this study was produced with a high amount of ligno-cellulose material, which is much more slowly degradable compared to ones containing no or little amount of such material. Similarly, to microbial biomass, microbial extracellular enzyme activity (EEA) can be modulated by changes induced by soil management (Vega-ávila et al., 2018). Enzymes play a key role in biochemical processes (Makoi, 2010; Das and Varma, 2011) and are considered as biochemical indices of soil quality; moreover, their activity is correlated with the microbial biomass and organic matter content (Gispert et al., 2013). In the present work, key enzymes responsible for the main nutrient cycles were analysed: β-glucosidase, chitinase (C), leucine-aminopeptidase (N), acid and alkaline phosphatase, pyrophosphate phosphodiesterase and phosphodiesterase (P) and arylsulfatase (S). Contrary to the observations of other authors, the activity of one of the key enzymes involved in the carbon cycle, β-glucosidase, was not influenced by the compost (Adetunji et al., 2017; Mondini et al., 2018). β-glucosidase is produced by by both bacteria and fungi in soil. The lack of increase of this enzyme is almost certainly related to the use of a mature compost, where the degradation of cellulose, requiring β-glucosidase as the enzyme in the final step, has almost been completed. In fact, β-glucosidase activity has been reported to peak in correspondence of the first phase of decomposition of organic substances, while in mature compost this enzyme has a lower activity (Papa et al., 2008).

Chitinase is a key enzyme responsible for breaking down and hydrolysing chitin and it is involved in the C cycle (Varma and Bakshi, 2011). In 2020, although no significant differences in chitinase activity were observed between treatments, a trend towards higher values was observed for Cmp. This trend was confirmed in the year 2021, when Cmp treatment showed significantly higher level of chitinase activity than Fert. The increase in chitinase activity might be related to the supply of substrates for this enzyme as well as to a higher turnover of fungi cell-wall, due to their growth, stimulated by compost addition. The increased concentration of chitin is also consistent with the higher abundance of Agaromycetes (**Supplementary Table 7**). As stated in the Šnajdr study (Šnajdr et al., 2009), soils with saprophytic Basidiomycetes raise chitinase activity. Further, this type of compost, with residues form different sources, provided an ideal source for the activity of this enzyme (Karaca et al., 2011; Tian et al., 2020). Leucyl aminopeptidase (LAP), an enzyme involved in the N cycle, breaks down organic matter, particularly specifically in breakdown of proteins. LAP activity increased in both treatments in the second and third year of study, showing significantly higher values for Cmp compared to Fert in 2021. This result is consistent with observations made by other authors and may be explained by an increased input of organic matter, serving as a source of nitrogen for soil microbes, as well as by an increased turnover of microbial proteins, due to increased microbial growth and activity (Asmar et al., 1994; Galvez et al., 2012). Four enzymes involved in biogeochemical cycle of P were tested: acid and alkaline phosphomonoesterase (ACP and ALP) and phosphodiesterase (PDE) together with pyrophosphate diesterase (PPase) were considered. ACP and ALP are thought to be involved in the mineralisation of P (Nannipieri et al., 2011), which plays a primary role in the nutritional requirements of plants. ALP enzyme is not synthesised by high plants and its activity is entirely up to microorganisms (Acosta-Martínez and Tabatabai, 2000). Our findings indicate that compost application induced significantly higher ACP and ALP activities in Cmp compared to Fert from the second year onward. As previous studies have shown, the addition of compost promotes phosphate mineralisation. This is due to compost composition, which is rich in organophosphorus compounds and provides a P source for microbes. PDE and PPases are also related to the P cycle and act on P diesters and pyrophosphate diesters respectively. As for other enzymes, no differences between treatments were found in the first year, while significantly higher levels of PPases and PDes were observed in Cmp treatment in 2020 and 2021. As reported in previous research, higher levels of these enzymes are associated with organic management, and particularly to manure application (Niemi et al., 2008; Chernysheva et al., 2018), It is worth noting that a higher activity of all tested enzymes related to the P cycle are improved, indicating an increased turnover of different organic P forms. It follows that biogeochemical P cycle is greatly stimulated in it different sub-cycle parts. This result is consistent with the results of our microbial taxonomic analysis. Cmp was characterised by an abundance of Firmicutes compared to Fert, which are positively correlated with P availability. As assumed by other authors, these bacteria can be placed in the category of phosphorus solubilizers (Das et al., 2017; Cui et al., 2019). This might explain the correlation between high ALP activity and high presence of Firmicutes in the Cmp treatment. Arylsulfatase (ARS) hydrolyses organic S esters and is therefore linked to the S cycle (Tabatabai and Bremmer, 1970). As for the other enzymes, differences in ARS activity between treatments could be detected from the second year onwards, with higher values for Cmp in 2020 and 2021. As found by (Karaca et al., 2011), compost is beneficial for the activity of this enzyme providing organosulphur compounds that microorganisms can easily attack. Further literature confirmed that increased organic matter and the consequent availability of sulphate esters promote ARS activity (Chang et al., 2007; Mackie et al., 2015; Malik et al., 2020).

Overall, in our trial compost application showed beneficial effects on the soil biological fertility, although improvement in the soil quality indicators we analysed were slower and more gradual than those observed in other studies (Bhattacharyya et al., 2005; Gianfreda and Ruggiero, 2006; Karaca et al., 2011; Lazcano et al., 2013). As confirmed by the soil chemical analysis performed in the third year of the study, the application of compost boosted nutrient turnover through both increased microbial biomass and enzyme activity, improving plant nutrient availability.

Soil fertilization practices has been shown to shape the microbial community composition and soil microbial diversity (Dinca et al., 2022). Considering their nutritional role, mineral fertilizers application directly conveys nutrients to plants, whereas organic fertilizers (*e.g*., manure, compost and digestates) are composed of complex molecules that are used by both plants and soil microbiota, thereby also increasing the soil organic matter content (Maeder et al., 2002). In this regard, the use of different type of fertilizers affects microbial growth and competitiveness because different bacterial and fungal groups can vary in their ability to use the different nutrient forms in soil (Dinca et al., 2022). Thus, with the aim of assessing whether the compost-based treatments over the three years experiment could have affected the composition of the microbiome inhabiting grapevine rhizosphere, bacterial and fungal communities were characterized.

Overall, the dominant bacterial phyla (Actinobacteriota, Proteobacteria, Acidobacteriota, and Firmicutes) identified in this study match with other studies that found those taxa to be dominant in grapevine rhizosphere (Darriaut et al., 2022). However, a selective enrichment of specific phyla was shifted by the fertilization practice, *i.e*., Actinobacteriota in Fert grapevines and Firmicutes in Cmp ones. The abundance of copiotrophic bacteria such as Proteobacteria, Firmicutes, and Actinobacteria in the soil can operate as a reliable indicator of soil health (Bhatti et al., 2017; Raiger Iustman et al., 2021). Moreover, several members of Actinobacteria phylum are known to improve the availability of nutrients and enhance the production of metabolites and promote plant growth regulators (Bhatti et al., 2017). Interestingly, the application of compost did not induce any significant alterations in both the α- and β-diversity of the bacterial community associated with grapevine rhizosphere. Despite this, LEfSe analysis indicated that *Firmicutes* was a biomarker in the rhizosphere of grapevines fertilized with compost. The enrichment of *Firmicutes* in the rhizosphere of agricultural plants can be indeed related to the root exudation activities and the bacteria belonging to this phylum have been widely characterized for their plant growth-promoting traits (Nwachukwu et al., 2021). In fact, these bacteria are generally associated with the ability of producing exopolysaccharide and siderophores, for P solubilization activity and for secretion of phytohormone-like molecules (e.g., indol-acetic acid) (Nwachukwu et al., 2021). In agreement with our observations, different studies have pointed out that either the partial or the complete substitution of mineral fertilizers with organic ones can lead to a significant enrichment of *Firmicutes* in both the bulk soil and in the rhizosphere of maize plants (Enebe and Babalola, 2020; Ren et al., 2021), possibly relating the adaptability of these bacterial phylum to the new fertilization strategy to their capability of decomposing the soil organic matter (Bolhuis et al., 2014).

Regarding the fungal groups, the detected microbial communities were dominantly composed of Ascomycota and Basidiomycota, as reported in previous studies on vineyard soils (Oyuela Aguilar et al., 2020; Darriaut et al., 2022). However, no significant changes at the phylum or class levels were triggered by the application of compost to grapevine soil. Ascomycota are generally the most dominant fungi found in soil systems globally, and their abundance may be related to their ability of utilizing a higher number of resources (Egidi et al., 2019). Several phylotypes of Ascomycota have been described to play fundamental roles in C and N cycling, soil stability, plant biomass decomposition, and endophytic interactions with plants (Challacombe et al., 2019). Interestingly, as also found for bacterial communities, the application of compost did not induce any significant change in the α-diversity of fungal communities, whereas variations in β-diversity were observed. In fact, the sole variation of the β-diversity within a vineyard can reflect the soil heterogeneity due to a possible turnover of soil microbial communities to fertilization practices without compromising the α-diversity values (Signorini et al., 2021). A deeper analysis at genus level showed that specific taxa were differentially enriched following the use of compost. *Diutina* and *Paracremonium* increased by the treatment with compost, whereas *Fusarium*, *Mycoleptodiscus*, *Plectosphaerella*, and *Papiliotrema* decreased. Members of the genera *Fusarium* and *Plectosphaerella* comprise several species of opportunistic plant pathogens affecting important agricultural crops (Nikitin et al., 2023). However, it has been reported that the amendment with organic fertilizers can reduce the relative abundance of these taxa and counteract the adverse effects of inorganic fertilizers (Ding et al., 2017). Additionally, LEfSe analysis pointed out members of *Saccharomycetales* and *Didymosphaeriaceae* as biomarkers in compost-treated soil. Some fungal communities associated with vineyard soils are able to transform insoluble minerals into a bioavailable form for plants. Indeed, the organic acid production by yeasts from *Saccharomycetales* taxon may contribute to inorganic P solubilization in the rhizosphere, where it can be taken up by roots (Mestre et al., 2011). Overall, our results showed that the organic fertilization with compost increased the abundance of beneficial fungi, whereas Fert soil was enriched with taxa encompassing plant pathogens. These results agree with other studies in which the addition of organic amendments largely suppressed different pathogenic taxa (Nikitin et al., 2023). Finally, it is important to highlight that the association between vine roots and arbuscular mycorrhizal fungi (AMF, from the Glomeromycota phylum) has been widely reported (Trouvelot et al., 2015). However, since the dominant taxa identified in this study was limited to the fungal taxa amplified with the primer pair herein used, there is the possibility of an underestimation of some fungal phylotypes that are poorly amplified with commonly used ITS primers, such as members of Glomeromycota (Oyuela Aguilar et al., 2020).

The relationship between vineyard performances and soil physiochemical and biological properties is not straightforward. Although fertilization is essential to sustain vine growth and production, application of organic amendments can drastically increase the capacity of the soil to supply nutrients, especially N, with a negative effect on grape and wine quality, due to excess vigor and high yields (Verdenal et al., 2021).

To monitor the N nutritional level in vines treated with compost or fertilizer, SPAD index was used every year during the growing season as an indicator of the foliar N content (Porro et al., 2001; Brunetto et al., 2012). Overall, SPAD values from both treatments were in the range of a regular N nutritional status; thus, it could be supposed that both treatments provided plants with a sufficient amount of available nitrate.

Interestingly, despite the higher rates of nitrogen supplied with compost, SPAD index didn’t show differences between treatments in the first two years of the experiment. Only after 3 consequent years of compost application SPAD indicated higher foliar N content in Cmp plants, in agreement with the higher N availability in the soil of this treatment revealed by the chemical analysis. These data suggest that compost application did not result in higher nitrogen uptake by the vines in the first years, potentially because of slow decomposition and mineralization rates of the SOM applied. Moreover, newly mineralized N might have been initially used by microorganisms, as attested by the greater microbial biomass and enzyme activity observed in Cmp treatment from the second year onward.

The vegetative growth measured on vines treated with compost or fertilizers was in agreement with the vine nutritional status. In fact, treatments showed similar values of canopy development in the first two years, while a significantly larger canopy area was found in Cmp treatment only in the third year, when SPAD were higher. Increased vine vigour following compost application was reported by other authors. (Pinamonti, 1998; Gaiotti et al., 2017) found higher pruning weights in vines treated with compost for 6 and 5 years, respectively. In their studies the vine response to compost supply was faster than in our trial, as growth improvements were already observed in the first year of application. To this regard, it must be noted that our trial was performed in a vineyard newly established on a soil disturbed by land displacements, thus recovering of soil chemical and biological fertility may have delayed the effects of amendment on vine growth.

Increased vine yields following compost application have been reported in several studies, being commonly attributed to increased nitrogen availability (Rubio et al., 2013; Gaiotti et al., 2017) or improved soil water status (Pinamonti, 1998; Ramos, 2017). In our study grape production was overall little affected by the application of compost, as we observed only a tendency towards slightly higher production in Cmp treatment. However, 2021 was the first productive season of our experimental vineyard, thus our results are not fully comparable to those commonly observed in established normally performing vineyards.

Concerning grape quality, sugar content was unaffected by the application of compost, while an increase in titratable acidity and malic acid content was observed in Cmp treatment. This could likely be attributed to the increased availability of exchangeable K observed in Cmp soil; in fact, potassium in berries has been shown to be correlated to malic acid content (Failla et al., 1996; Marcuzzo et al., 2021). While previous research suggested that increased K supply from compost application may also increase wine pH (Lazcano et al., 2020) this negative effect was not observed in our study; on the contrary, a slight but significant reduction of must pH was observed in Cmp musts. Although, in the first year of production, we observed moderate improvements in grape quality derived from the use of compost, additional benefits can be expected in the medium and long term on fully productive vines.

## Conclusions

To the best of our knowledge, this is the first study that investigated the use of compost in vineyard by means of a multifactorial approach, in the attempt to depict interrelated dynamic effects between soil properties, microbial community structure and function and plant nutritional status. The study was performed on a vineyard whose soil was highly disturbed by land terracing operations before plantation. In this specific context, compost application had the double purpose of restoring the soil fertility while supplying nutrients to the vines, improving the traditional vine nutrition management performed with chemical fertilizers.

Our results show that the application of compost for three consequent years significantly increased the SOM and improved nutrient turnover, with a general enrichment of nutrients readily available to the plants.

Soil biological fertility, assessed by quality indicators such as the microbial biomass and the enzyme activity, was positively affected by the compost treatment, but more gradually than what observed in other studies, as improvements were mainly observed from the second year onward. Organic fertilization induced changes in the soil microbial community structure, promoting the presence of copiotrophic bacteria, indicators of soil quality, and solubilizing phosphorus soil bacteria. A reduction of fungal pathogenic strains was also observed, suggesting a positive effect of compost application on soil health. Combined analysis of the data of microbial community structure, enzyme activity, soil chemical analysis and vine nutritional index showed interrelated effects of compost on these parameters. Indeed, compost application resulted in a general improvement of soil chemical and biological properties in the mid-term, that was directly related to an increase in plant nutrient uptake and vegetative growth, compared to those observed in chemically fertilized vines. In the first year of production, grape yield and quality were affected to a small extent by compost addition; however, the trend toward increased yield and the improvement of some grape parameters such as the acidity and pH suggest that, on fully productive vines, compost might guarantee agronomic performances comparable or even improved than those of chemically fertilized vines.

Overall, our findings provide evidence that compost can improve the recovery of soil functionality and biodiversity in young vineyards disturbed by land terracing, preventing the loss of soil characteristics strictly related to the expression of the terroir. Further research is needed to better link changes in soil microbial structure and below-ground ecosystem processes induced by the application of compost. A deeper understanding of release timing of available nutrients from mineralization of organic inputs will be essential for a more efficient management of organic fertilization.

## Data availability statement

The data presented in study are deposited in the NCBI SRA, accession number PRJNA1023065.

## Author contributions

ML: Formal Analysis, Investigation, Methodology, Visualization, Writing – original draft, Writing – review & editing. AR: Data curation, Formal Analysis, Software, Writing – original draft. MA: Formal Analysis, Investigation, Software, Writing – original draft. FF: Formal Analysis, Investigation, Writing – review & editing. SM: Formal Analysis, Writing – review & editing. YP: Data curation, Formal Analysis, Investigation, Methodology, Software, Writing – original draft. PM: Validation, Writing – review & editing. LL: Data curation, Software, Writing – review & editing. FG: Conceptualization, Data curation, Investigation, Methodology, Project administration, Resources, Supervision, Validation, Visualization, Writing – original draft, Writing – review & editing.
