# Supplementary material for: Compost application boosts soil restoration in highly disturbed hillslope vineyard
Source: Front Plant Sci. 2023 Nov 23;14:1289288. doi: 10.3389/fpls.2023.1289288 (PMC10702515; doi:10.3389/fpls.2023.1289288)
Supplement: Supplementary file 1 [file DataSheet_1.docx]

**Supplementary material:**

**Tables**

**Supplementary Table 1** Enzyme measured in soil sample through the three-year’s study and throughout the season. Cmp (compost), Fert (fertilised). Enzymes units of measure Hymecromone nmol per g of soil per h. Leucyl aminopeptidase units of measure C₁₀H₉NO₂ nmol per g of soil per h.

|  | | **2019** | | | | | **2020** | | | | **2021** | | | |
| --- | --- | --- | --- | --- | --- | --- | --- | --- | --- | --- | --- | --- | --- | --- |
|  | **April** | | | **October** | | | **April** | | **October** | | **April** | | **October** | |
|  | **Cmp** | | **Fert** | **Cmp** | | **Fert** | **Cmp** | **Fert** | **Cmp** | **Fert** | **Cmp** | **Fert** | **Cmp** | **Fert** |
| **β-Glucosidase** | 6.1 | | 10.8 | 2.1 | | 2.7 | 9.2 | 11.9 | 10.1 | 8.2 | 8.9 | 7.7 | 7.2 | 7.0 |
| **Chitinase** | 5.5 | | 10.1 | 1.9 | | 2.6 | 11.5 | 10.0 | 12.3 | 8.6 | 14,6 a | 11,1 b | 18,4 a | 11,6 b |
| **Leucyl aminopeptidase** | 31.9 | | 52.6 | 12.7 | | 16.8 | 246.8 | 259.3 | 275.6 | 193.6 | 89.9 | 78.2 | 96.4 | 79.8 |
| **Acid phosphatase** | | 52.6 | 104.3 | 18.1 | | 31.1 | 3.77 | 33.9 | 54,7 a | 39,9 b | 72,5 a | 59,8 b | 78,3 a | 63,9 b |
| A**lkaline phosphatase** | | 177.6 | 325.5 | 90.1 | | 132.9 | 1178 a | 982 b | 1485 a | 1003 b | 771.0 a | 410,5 b | 689.9 | 541.4 |
| **Pyrophosphate** - P**hosphodiesterase** | | 17.3 | 39.5 | 9.9 | | 15.9 | 9,3 a | 7,4 b | 13,6 a | 9,3 b | 20.0 | 16.7 | 20,7 a | 17,2 b |
| P**hosphodiesterase** | | 38.1 | 68.2 | 25.4 | | 40.4 | 140 a | 103 b | 190 a | 119 b | 102,3 a | 74,1 b | 102,3 a | 80,6 b |
| **Arylsulfatase** | | 37.2 | 70.9 | 13.6 | 20.4 | | 17,3 a | 12,5 b | 25,9 a | 18,3 b | 30,9 a | 21,1 b | 30.0 a | 26,3 b |

**Supplementary Table 2. Sequencing and sequence processing results.** Raw PE means the raw PE reads after sequencing; Combined are the tags sequences obtained by splicing; Qualified are the sequences after Raw Tags performed filtering low quality and short length; Nochime are the Tag sequences filtered the chimera, that is Effective Tags which are finally used for subsequent analysis.

|  | **Sample Name** | **Raw PE(#)** | **Combined(#)** | **Qualified(#)** | **Nochime(#)** |
| --- | --- | --- | --- | --- | --- |
| 16S | Cmp1 | 31,804 | 28,431 | 28,232 | 19,120 |
|  | Cmp2 | 39,366 | 33,916 | 33,683 | 20,671 |
|  | Cmp3 | 34,174 | 23,682 | 23,485 | 16,943 |
|  | Cmp4 | 40,324 | 34,722 | 34,444 | 20,742 |
|  | Fert1 | 32,469 | 29,240 | 28,995 | 18,700 |
|  | Fert2 | 41,540 | 34,912 | 34,576 | 23,029 |
|  | Fert3 | 42,064 | 37,090 | 36,754 | 22,140 |
|  | Fert4 | 46,709 | 36,377 | 36,065 | 25,036 |
| ITS2 | Cmp1 | 46,188 | 44,666 | 44,559 | 37,325 |
|  | Cmp2 | 39,961 | 38,102 | 38,001 | 30,861 |
|  | Cmp3 | 38,891 | 37,010 | 36,546 | 30,030 |
|  | Cmp4 | 46,582 | 44,702 | 44,573 | 36,008 |
|  | Fert1 | 45,031 | 42,083 | 41,795 | 33,422 |
|  | Fert2 | 46,590 | 44,064 | 43,875 | 36,271 |
|  | Fert3 | 44,431 | 42,943 | 42,833 | 36,128 |
|  | Fert4 | 38,367 | 36,352 | 36,244 | 30,217 |

**Supplementary Table 3. Relative abundance of bacterial taxa at phylum level.**

|  |  | **Cmp** | | |  | **Fert** | | |
| --- | --- | --- | --- | --- | --- | --- | --- | --- |
| **Phylum** | **N** | **mean** | **sd** | **se** |  | **mean** | **sd** | **se** |
|  |  |  |  |  |  |  |  |  |
| Acidobacteriota | 4 | 11.63 | 2.70 | 1.35 |  | 12.99 | 1.46 | 0.73 |
| Actinobacteriota | 4 | 27.80 | 7.31 | 3.65 |  | 34.27 | 3.81 | 1.90 |
| Bacteroidota | 4 | 2.73 | 0.75 | 0.38 |  | 2.39 | 0.34 | 0.17 |
| Chloroflexi | 4 | 3.94 | 0.58 | 0.29 |  | 4.57 | 0.08 | 0.04 |
| Firmicutes | 4 | 14.10 | 6.50 | 3.25 |  | 4.08 | 0.78 | 0.39 |
| Gemmatimonadota | 4 | 2.97 | 0.33 | 0.16 |  | 4.15 | 0.73 | 0.36 |
| Myxococcota | 4 | 2.01 | 0.64 | 0.32 |  | 2.12 | 0.17 | 0.09 |
| Nitrospirota | 4 | 2.50 | 1.05 | 0.53 |  | 2.23 | 0.20 | 0.10 |
| Other | 4 | 5.67 | 1.65 | 0.83 |  | 6.78 | 0.85 | 0.43 |
| Proteobacteria | 4 | 26.66 | 3.74 | 1.87 |  | 26.43 | 4.24 | 2.12 |

**Supplementary Table 4. Relative abundance of bacterial taxa at class level, within Acidobacteriota and Actinobacteriota phyla.**

|  |  |  | **Cmp** | | |  | **Fert** | | |
| --- | --- | --- | --- | --- | --- | --- | --- | --- | --- |
| **Phylum** | **Class** | **N** | **mean** | **sd** | **se** |  | **mean** | **sd** | **se** |
|  |  |  |  |  |  |  |  |  |  |
| Acidobacteriota | Acidobacteriae | 4 | 8.08 | 1.766 | 0.88 |  | 7.44 | 1.48 | 0.74 |
| Acidobacteriota | Blastocatellia | 4 | 19.97 | 4.50 | 2.25 |  | 20.07 | 1.95 | 0.97 |
| Acidobacteriota | Holophagae | 4 | 1.93 | 0.48 | 0.24 |  | 3.50 | 1.20 | 0.60 |
| Acidobacteriota | Others | 4 | 6.31 | 0.76 | 0.38 |  | 5.87 | 1.14 | 0.57 |
| Acidobacteriota | Thermoanaerobaculia | 4 | 3.87 | 2.30 | 1.15 |  | 4.45 | 2.06 | 1.03 |
| Acidobacteriota | Vicinamibacteria | 4 | 59.85 | 2.01 | 1.00 |  | 58.67 | 2.86 | 1.43 |
| Actinobacteriota | Acidimicrobiia | 4 | 11.98 | 2.55 | 1.27 |  | 8.87 | 1.03 | 0.51 |
| Actinobacteriota | Actinobacteria | 4 | 61.42 | 4.85 | 2.42 |  | 57.67 | 6.97 | 3.487 |
| Actinobacteriota | MB-A2-108 | 4 | 4.392 | 0.94 | 0.47 |  | 6.99 | 1.74 | 0.87 |
| Actinobacteriota | Others | 4 | 2.43 | 0.45 | 0.23 |  | 3.49 | 0.22 | 0.11 |
| Actinobacteriota | Thermoleophilia | 4 | 19.77 | 3.11 | 1.55 |  | 22.97 | 4.71 | 2.35 |

**Supplementary Table 5. Relative abundance of bacterial taxa at order level, within the classes Alphaproteobacteria and Gammaproteobacteria**.

|  |  |  |  | **Cmp** | | |  | **Fert** | | |
| --- | --- | --- | --- | --- | --- | --- | --- | --- | --- | --- |
| **Phylum** | **Class** | **Order** | **N** | **mean** | **sd** | **se** |  | **mean** | **sd** | **se** |
|  |  |  |  |  |  |  |  |  |  |  |
| Proteobacteria | Alphaproteobacteria | Acetobacterales | 4 | 0.93 | 0.42 | 0.21 |  | 0.80 | 0.46 | 0.23 |
| Proteobacteria | Alphaproteobacteria | Azospirillales | 4 | 3.27 | 1.22 | 0.61 |  | 6.46 | 0.24 | 0.12 |
| Proteobacteria | Alphaproteobacteria | Caulobacterales | 4 | 2.13 | 0.59 | 0.29 |  | 2.31 | 0.85 | 0.43 |
| Proteobacteria | Alphaproteobacteria | Defluviicoccales | 4 | 5.66 | 2.47 | 1.23 |  | 2.18 | 1.66 | 0.83 |
| Proteobacteria | Alphaproteobacteria | Dongiales | 4 | 2.46 | 0.24 | 0.12 |  | 2.99 | 0.41 | 0.20 |
| Proteobacteria | Alphaproteobacteria | Micropepsales | 4 | 0.26 | 0.21 | 0.10 |  | 0.12 | 0.02 | 0.01 |
| Proteobacteria | Alphaproteobacteria | Others | 4 | 4.07 | 0.67 | 0.33 |  | 3.35 | 1.02 | 0.51 |
| Proteobacteria | Alphaproteobacteria | Reyranellales | 4 | 1.43 | 0.60 | 0.30 |  | 1.95 | 0.32 | 0.16 |
| Proteobacteria | Alphaproteobacteria | Rhizobiales | 4 | 69.43 | 5.52 | 2.76 |  | 66.74 | 1.89 | 0.95 |
| Proteobacteria | Alphaproteobacteria | Rhodobacterales | 4 | 1.02 | 0.32 | 0.16 |  | 1.40 | 0.76 | 0.38 |
| Proteobacteria | Alphaproteobacteria | Rhodospirillales | 4 | 0.24 | 0.10 | 0.05 |  | 0.19 | 0.06 | 0.03 |
| Proteobacteria | Alphaproteobacteria | Sphingomonadales | 4 | 5.27 | 0.60 | 0.30 |  | 7.29 | 0.75 | 0.38 |
| Proteobacteria | Alphaproteobacteria | Tistrellales | 4 | 3.82 | 1.15 | 0.58 |  | 4.21 | 1.49 | 0.75 |
| Proteobacteria | Gammaproteobacteria | Burkholderiales | 4 | 52.86 | 4.12 | 2.06 |  | 50.82 | 3.06 | 1.53 |
| Proteobacteria | Gammaproteobacteria | Chromatiales | 4 | 4.29 | 3.79 | 1.89 |  | 3.18 | 3.17 | 1.59 |
| Proteobacteria | Gammaproteobacteria | Competibacterales | 4 | 1.79 | 1.77 | 0.88 |  | 0.86 | 1.19 | 0.59 |
| Proteobacteria | Gammaproteobacteria | Enterobacterales | 4 | 11.46 | 13.27 | 6.64 |  | 2.50 | 1.27 | 0.63 |
| Proteobacteria | Gammaproteobacteria | Others | 4 | 15.00 | 5.14 | 2.57 |  | 13.19 | 1.33 | 0.67 |
| Proteobacteria | Gammaproteobacteria | PLTA13 | 4 | 4.10 | 1.62 | 0.81 |  | 4.35 | 1.44 | 0.72 |
| Proteobacteria | Gammaproteobacteria | Pseudomonadales | 4 | 2.77 | 1.67 | 0.84 |  | 1.23 | 1.25 | 0.63 |
| Proteobacteria | Gammaproteobacteria | Steroidobacterales | 4 | 5.20 | 2.28 | 1.14 |  | 13.07 | 3.06 | 1.53 |
| Proteobacteria | Gammaproteobacteria | Xanthomonadales | 4 | 2.53 | 3.93 | 1.96 |  | 10.80 | 7.32 | 3.66 |

**Supplementary Table 6. Relative abundance of fungal taxa at phylum level.**

|  |  | **Cmp** | | |  | **Fert** | | |
| --- | --- | --- | --- | --- | --- | --- | --- | --- |
| **Phylum** | **N** | **mean** | **sd** | **se** |  | **mean** | **sd** | **se** |
|  |  |  |  |  |  |  |  |  |
| Aphelidiomycota | 4 | 0.02 | 0.03 | 0.01 |  | 0.05 | 0.01 | 0.01 |
| Ascomycota | 4 | 78.20 | 4.91 | 2.46 |  | 72.87 | 9.64 | 4.82 |
| Basidiomycota | 4 | 1.2 | 0.46 | 0.23 |  | 2.02 | 0.60 | 0.30 |
| Chytridiomycota | 4 | 0.01 | 0.01 | 0.01 |  | 0.01 | 0.01 | 0.01 |
| Glomeromycota | 4 | 0.01 | 0.02 | 0.001 |  | 0.01 | 0.011 | 0.01 |
| Kickxellomycota | 4 | 0.04 | 0.02 | 0.01 |  | 0.06 | 0.03 | 0.01 |
| Monoblepharomycota | 4 | 0.02 | 0.02 | 0.01 |  | 0.01 | 0.01 | 0.01 |
| Mortierellomycota | 4 | 0.36 | 0.29 | 0.15 |  | 0.66 | 0.23 | 0.12 |
| Mucoromycota | 4 | 0.01 | 0.02 | 0.01 |  | 0.00 | 0.00 | 0.00 |
| Rozellomycota | 4 | 0.65 | 0.58 | 0.29 |  | 0.43 | 0.53 | 0.26 |
| unassigned | 4 | 19.49 | 5.59 | 2.80 |  | 23.93 | 10.32 | 5.16 |

**Supplementary Table 7. Relative abundance of fungal taxa at class level, within Ascomycota and Basidiomycota phyla.**

|  |  |  | **Cmp** | | |  | **Fert** | | |
| --- | --- | --- | --- | --- | --- | --- | --- | --- | --- |
| **Phylum** | **Class** | **N** | **mean** | **sd** | **se** |  | **mean** | **sd** | **se** |
|  |  |  |  |  |  |  |  |  |  |
| Ascomycota | Dothideomycetes | 4 | 20.70 | 16.81 | 8.40 |  | 24.02 | 5.76 | 2.88 |
| Ascomycota | Eurotiomycetes | 4 | 3.66 | 2.55 | 1.28 |  | 2.37 | 1.02 | 0.51 |
| Ascomycota | Laboulbeniomycetes | 4 | 0.01 | 0.02 | 0.01 |  | 0.01 | 0.01 | 0.01 |
| Ascomycota | Leotiomycetes | 4 | 3.77 | 6.93 | 3.47 |  | 0.64 | 0.40 | 0.20 |
| Ascomycota | Orbiliomycetes | 4 | 0.03 | 0.02 | 0.01 |  | 0.09 | 0.04 | 0.02 |
| Ascomycota | Pezizomycetes | 4 | 2.02 | 2.97 | 1.49 |  | 0.28 | 0.14 | 0.07 |
| Ascomycota | Saccharomycetes | 4 | 12.02 | 4.19 | 2.09 |  | 6.50 | 2.84 | 1.42 |
| Ascomycota | Sordariomycetes | 4 | 56.82 | 17.65 | 8.83 |  | 65.39 | 6.65 | 3.33 |
| Ascomycota | Unassigned | 4 | 0.97 | 0.69 | 0.34 |  | 0.71 | 0.44 | 0.22 |
| Basidiomycota | Agaricomycetes | 4 | 45.86 | 26.74 | 13.37 |  | 15.55 | 12.91 | 6.46 |
| Basidiomycota | Malasseziomycetes | 4 | 0.00 | 0.00 | 0.00 |  | 0.39 | 0.78 | 0.39 |
| Basidiomycota | Microbotryomycetes | 4 | 0.29 | 0.59 | 0.29 |  | 1.85 | 1.07 | 0.53 |
| Basidiomycota | Tremellomycetes | 4 | 49.51 | 25.65 | 12.82 |  | 81.82 | 12.14 | 6.07 |
| Basidiomycota | Unassigned | 4 | 4.38 | 3.67 | 1.83 |  | 0.40 | 0.79 | 0.40 |

**Figures:**


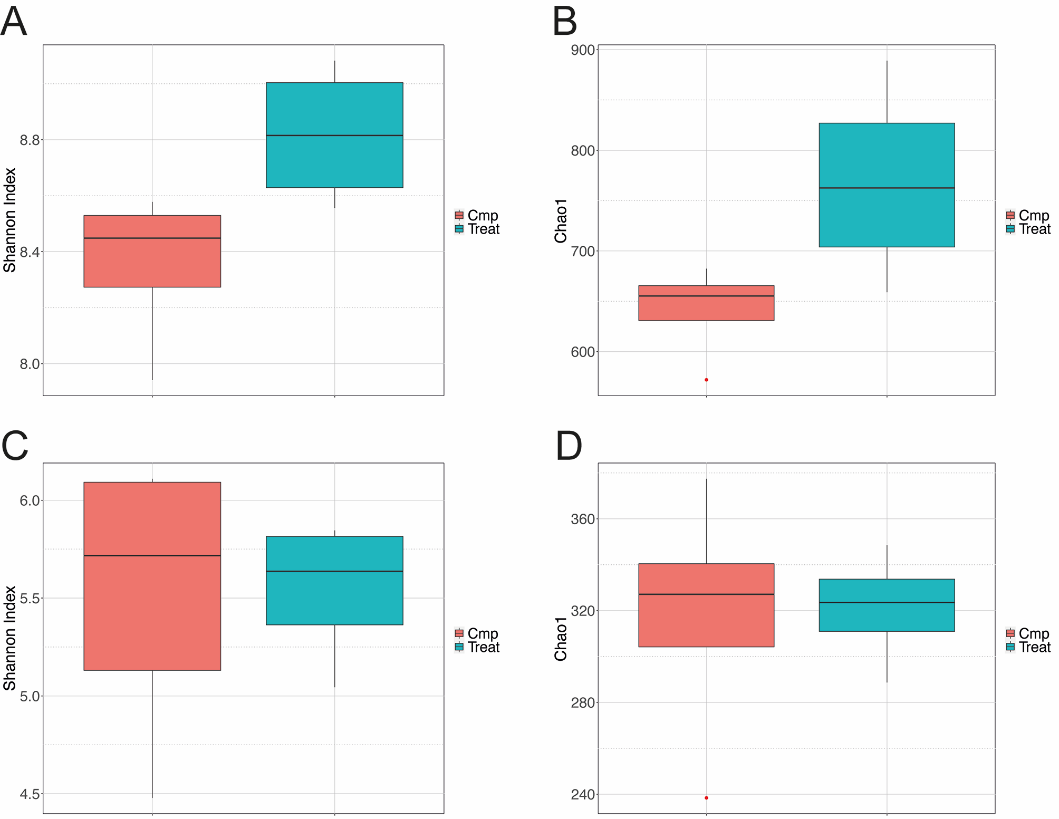


**Supplementary Figure 1. Boxplot of alpha diversity indexes. A.** Alpha diversity Shannon index of bacterial community. **B.** Alpha diversity Chao1 index of bacterial community. **C.** Alpha diversity Shannon index of fungal community. **D.** Alpha diversity Chao1 index of fungal community. No significant differences observed for the applied treatments upon Kruskal-Wallis test followed by Dunn's post-hoc test.


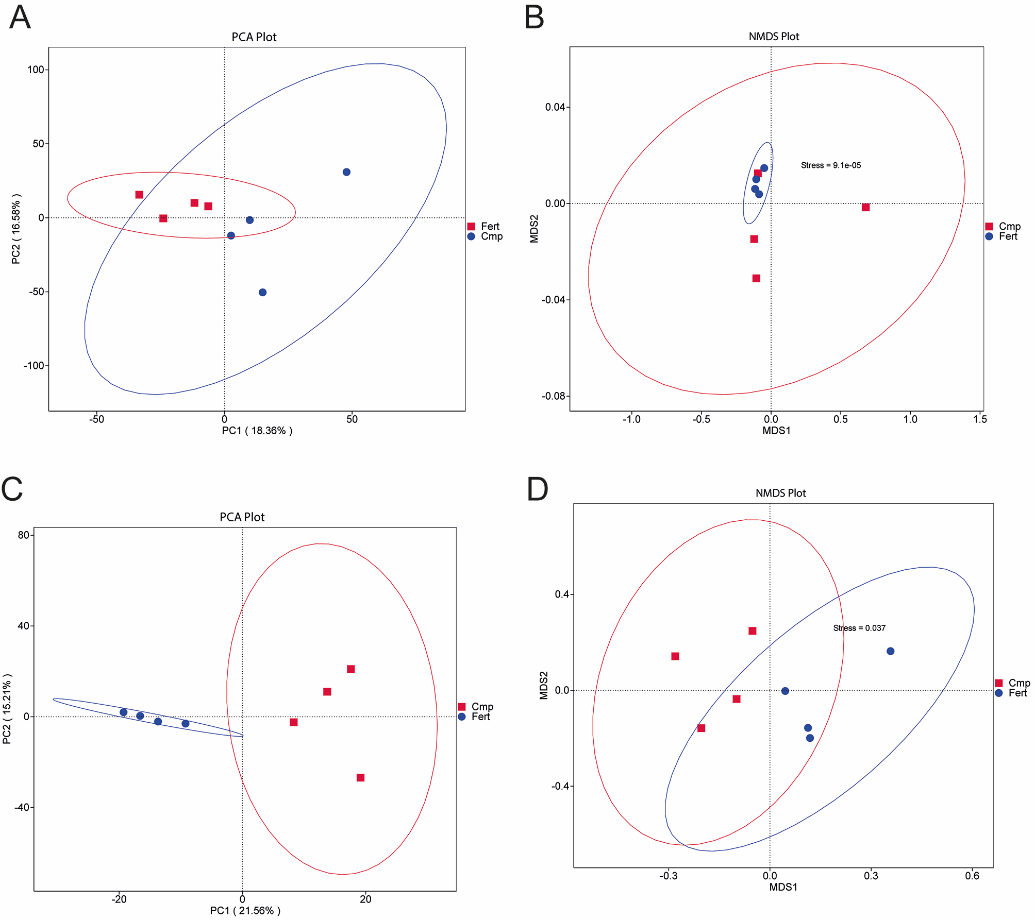


**Supplementary Figure 2. Beta diversity analysis to estimate the dissimilarity and similarity of microbial communities composition among different samples. A.** Principal component analysis (PCA) depicting the diversity of bacterial community. **B.** Non-metric multi-dimensional scaling analysis (NMDS) based on Weighted UniFrac distance matrix depicting the diversity of bacterial community. **C.** Principal component analysis (PCA) depicting the diversity of fungal community. **D.** Non-metric multi-dimensional scaling analysis (NMDS) based on Weighted UniFrac distance matrix depicting the diversity of fungal community.
